# Allostery of DNA nanostructures controlled by enzymatic modifications

**DOI:** 10.1093/nar/gkaa488

**Published:** 2020-06-11

**Authors:** Qi Yan, Yaqi Wang, Jile Shi, Bryan Wei

**Affiliations:** School of Life Sciences, Tsinghua University–Peking University Center for Life Sciences, Center for Synthetic and Systems Biology, Tsinghua University, Beijing 100084, China; School of Life Sciences, Tsinghua University–Peking University Center for Life Sciences, Center for Synthetic and Systems Biology, Tsinghua University, Beijing 100084, China; School of Life Sciences, Tsinghua University–Peking University Center for Life Sciences, Center for Synthetic and Systems Biology, Tsinghua University, Beijing 100084, China; School of Life Sciences, Tsinghua University–Peking University Center for Life Sciences, Center for Synthetic and Systems Biology, Tsinghua University, Beijing 100084, China

## Abstract

Allostery is comprehensively studied for natural macromolecules, such as proteins and nucleic acids. Here, we present controllable allostery of synthetic DNA nanostructure–enzyme systems. Rational designs of the synthetic allosteric systems are based on an in-depth understanding of allosteric sites with several types of strand placements, whose varying stacking strengths determine the local conformation and ultimately lead to a gradient level of allosteric transition. When enzymes in a molecular cloning toolbox such as DNA polymerase, exonuclease and ligase are applied to treat the allosteric sites, the resulting local conformational changes propagate through the entire structure for a global allosteric transition.

## INTRODUCTION

Rotation about a certain single bond can result in different conformations for a small organic molecule. In biomacromolecules and especially proteins, local chemical bond rotation can cascade across a long chain of chemical bonds to result in a conformational change at a distant site of the molecule. Such a phenomenon of allostery and the corresponding biological functions are well documented (1–3). Moreover, allostery of DNA–protein complexes also reveals the mechanistic details of some important biochemical and biomolecular processes (4,5). Our synthetic systems are based on nanostructures constructed by a scaffolded DNA origami method, which entails a long ‘scaffold’ DNA strand to fold into a complex shape with complementation of hundreds of short ‘staple’ strands (6–11). According to several recent studies, allostery can be designed in synthetic DNA systems (12–16). Especially, the inclusion or exclusion of effector strands at allosteric sites leads to a controllable allostery for DNA nanostructures (14,15).

What we present in this study are synthetic DNA nanostructure–enzyme systems, whose allostery is controlled by enzymatic modifications on allosteric sites. In the cases of DNA polymerase treatment, when the void allosteric sites are filled by templated elongation, local stacking orientation preference cascades to a global conformational change. Such a controllable allosteric transition based on DNA polymerase treatment is demonstrated for several unique structures. Then, we show that different types of effector staples at allosteric sites have different base stacking strengths. Based on the ranking of stacking strengths, we manage to enzymatically modify one type of effector staples into another type to achieve controllable allostery. For example, exonuclease is used to trim overhangs of effector staples, and ligase is used to join effector staples with nicks in between. Besides common continuous effector staples, other types of effector staples can be enzymatically modified from void allosteric sites in order to initiate the desired allostery. In summary, enzymatically controllable allostery is showcased as a general scheme for different enzymatic processes in a few unique DNA nanostructures.

## MATERIALS AND METHODS

### Sequence design

The DNA strands were generated by caDNAno and synthesized by Bioneer Corporation. Scaffold strand M13mp18 and M13-based vector of length 8064 bases (p8064) (8) were used.

### Structural self-assembly

Every staple strand (50 nM) and scaffold strand (10 nM) were mixed in one of three types of buffers: (A) 0.5× TE buffer (5 mM Tris, 1 mM EDTA) with 10–30 mM MgCl_2_ (pH 8); (B) 1× T4 ligase buffer (50 mM Tris–HCl, 10 mM MgCl_2_, 1 mM ATP, 10 mM DTT, pH 7.5, at 25°C) (NEB); and (C) 0.75× Exonuclease VII reaction buffer (37.5 mM Tris–HCl, 37.5 mM sodium phosphate, 6 mM EDTA, 7.5 mM 2-mercaptoethanol, pH 8, at 25°C) (NEB). Buffer C was applicable to the tests of stacking strength ranking of different types of effector staples. The mixture was subjected to a thermal annealing protocol: 94°C for 3 min followed by a rapid ramp from 90 to 60°C (5 min per °C) and a slow ramp from 60 to 25°C (25 min per °C).

### Enzymatic modifications

The annealed DNA samples were modified by enzymes in different reaction systems. (i) Q5 DNA polymerase: 9 μl DNA sample in buffer A was mixed with 9 μl Q5 High-Fidelity 2× Master Mix (or 9 μl 2× Q5 Reaction Buffer as in a negative control) (NEB) for an incubation at 37°C for 5 h. (ii) T4 DNA ligase: 1 μl of T4 ligase was mixed with 20 μl annealed DNA sample in buffer B for an incubation at 16°C for 17 h. Effector staples were subjected to phosphorylation by T4 PNK (NEB) before ligation treatment.

### Gel electrophoresis and purification

Annealed DNA samples (with or without enzymatical modifications) were purified by agarose gel electrophoresis (1.5–2%) before atomic force microscopy (AFM)/transmission electron microscopy (TEM) imaging. Gels were run in 0.5× TBE buffer (45 mM Tris, 1 mM EDTA, 45 mM boric acid) with 10 mM MgCl_2_ and stained with SYBR Safe (Thermo Fisher Scientific). Target bands were excised under blue light and crushed in Freeze’N Squeeze columns (Bio-Rad) and then directly subjected to centrifugation at 1000 *g* for 2 min at 4°C.

### AFM imaging

The morphology of the DNA structures was characterized by AFM (Multimode 8, Bruker) in liquid ScanAsyst mode. A 2 μl droplet of sample (1–10 nM, purified or unpurified) and a 50 μl drop of 0.5× TE buffer with 10 mM MgCl_2_ were applied to a freshly cleaved mica surface.

### TEM imaging

The morphology of 3D structures was characterized by TEM. One milliliter of 2% aqueous uranyl formate was mixed with 5 μl of 5 M NaOH and centrifuged at 14 000 *g* for 10 min to serve as the stain solution. A 10 μl droplet (2–10 nM) of purified sample was pipetted onto the glow-discharged, carbon-coated grid (Electron Microscopy Sciences) for 4 min and then wicked off and stained for 5 s with 4 μl of stain solution. The stain solution was then blotted off by filter paper and left on the grid to air-dry. The stained sample was analyzed on FEI Tecnai Spirit, operated at 120 kV at 26 000–63 000× magnification.

## RESULTS

**Figure 1.**
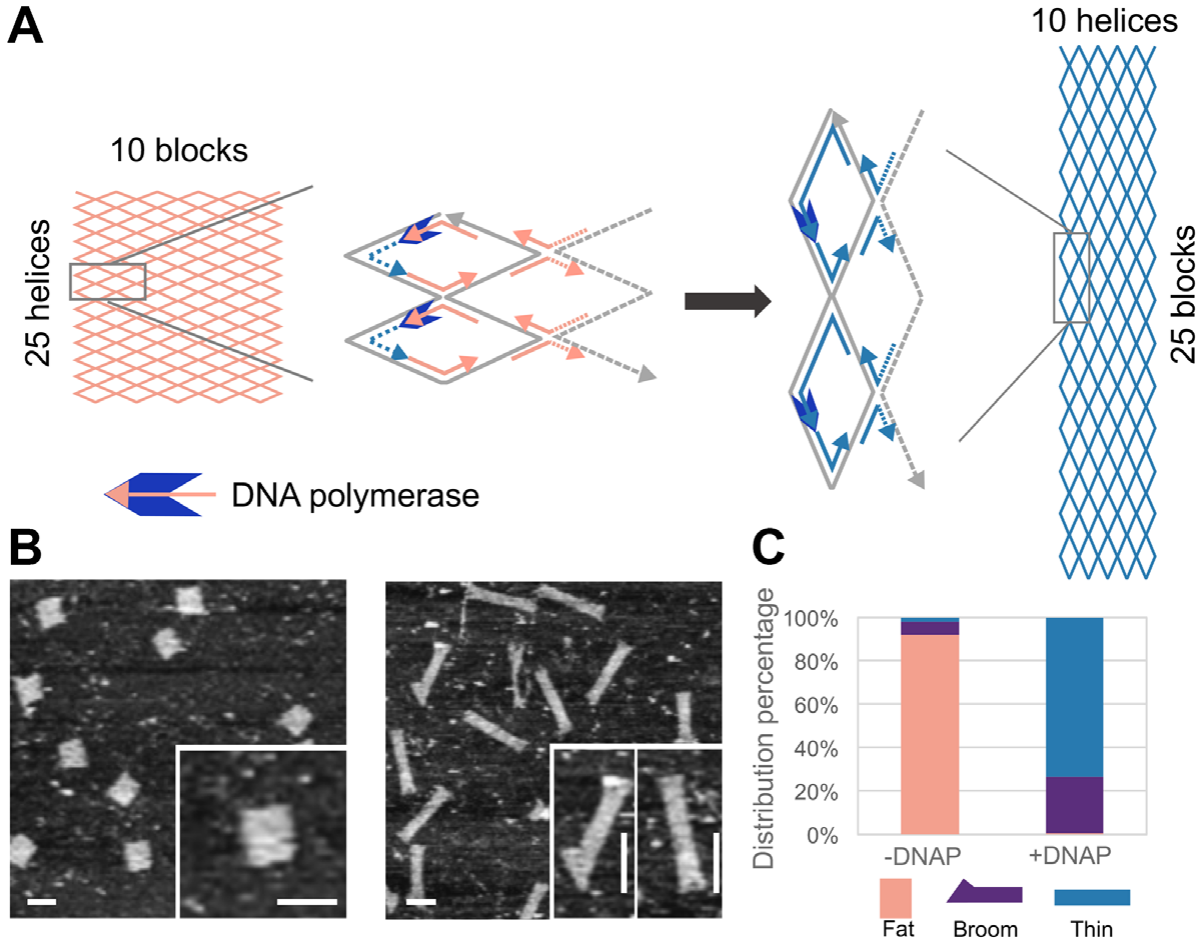
Allostery of a DNA nanostructure–DNA polymerase system. (**A**) Schematics of allostery based on enzymatic gap filling. The local gap filling at vertical boundary allosteric sites by DNA polymerase leads to the global allosteric transition from a fat rectangle to a thin rectangle (diagram of the gradual transition in Supplementary Figure S3). (**B**) AFM results without (left) and with (right) DNA polymerase treatment. Zoomed-in views are shown in insets. (**C**) Distribution of allosteric states without (*N* = 182) and with (*N* = 279) DNA polymerase treatment. Scale bars: 100 nm.

As a case study, a DNA nanostructure adapted from an earlier work was used (15). Folded without allosteric effector staples, the origami structure took a fat rectangle configuration (10*B* × 25*H* containing 10 blocks with 25 parallel helices in each block), which was measured at 76 ± 7 nm × 92 ± 8 nm (*N* = 30) under AFM (Figure 1A and B, left; details in Supplementary Figures S1 and S2). When effector staples were hybridized to fill the allosteric sites in a positive control, local conformational switch propagated through for a global structural reconfiguration (Supplementary Figure S1). Instead of hybridizing effector staples to void allosteric sites, we sought to accomplish the gap filling task by enzymatic elongation (17). When DNA polymerase-based elongation was applied to fill the void allosteric sites, the local conformational preference cascaded from the vertical boundary junctions to the entire origami structure, resulting in an allosteric transition from a fat rectangle (10*B* × 25*H*) to a thin rectangle (10*H* × 25*B*), which was measured at 37 ± 4 nm × 222 ± 11 nm (*N* = 30) under AFM (Figure 1A and B, right; details in Supplementary Figures S2 and S4). Besides the two typical rectangular configurations, a broom-like configuration was presented as an intermediate allosteric state, which was also identifiable under AFM (14,15). Before polymerase treatment, the distribution of three allosteric states (fat rectangle, broom and thin rectangle) was 92%, 6% and 2%, and after DNA polymerase treatment at 37°C for 5 h, the distribution became 1%, 26% and 73%, respectively (Figure 1C; Supplementary Figures S4 and S5). Q5 DNA polymerase was chosen for the gap filling task, and other enzymes such as T4 DNA polymerase were also tested, but the results were less satisfactory (Supplementary Figure S4). The results clearly demonstrated that the templated elongation at allosteric sites by DNA polymerase was effective for allosteric transition (Supplementary Figure S6).

**Figure 2.**
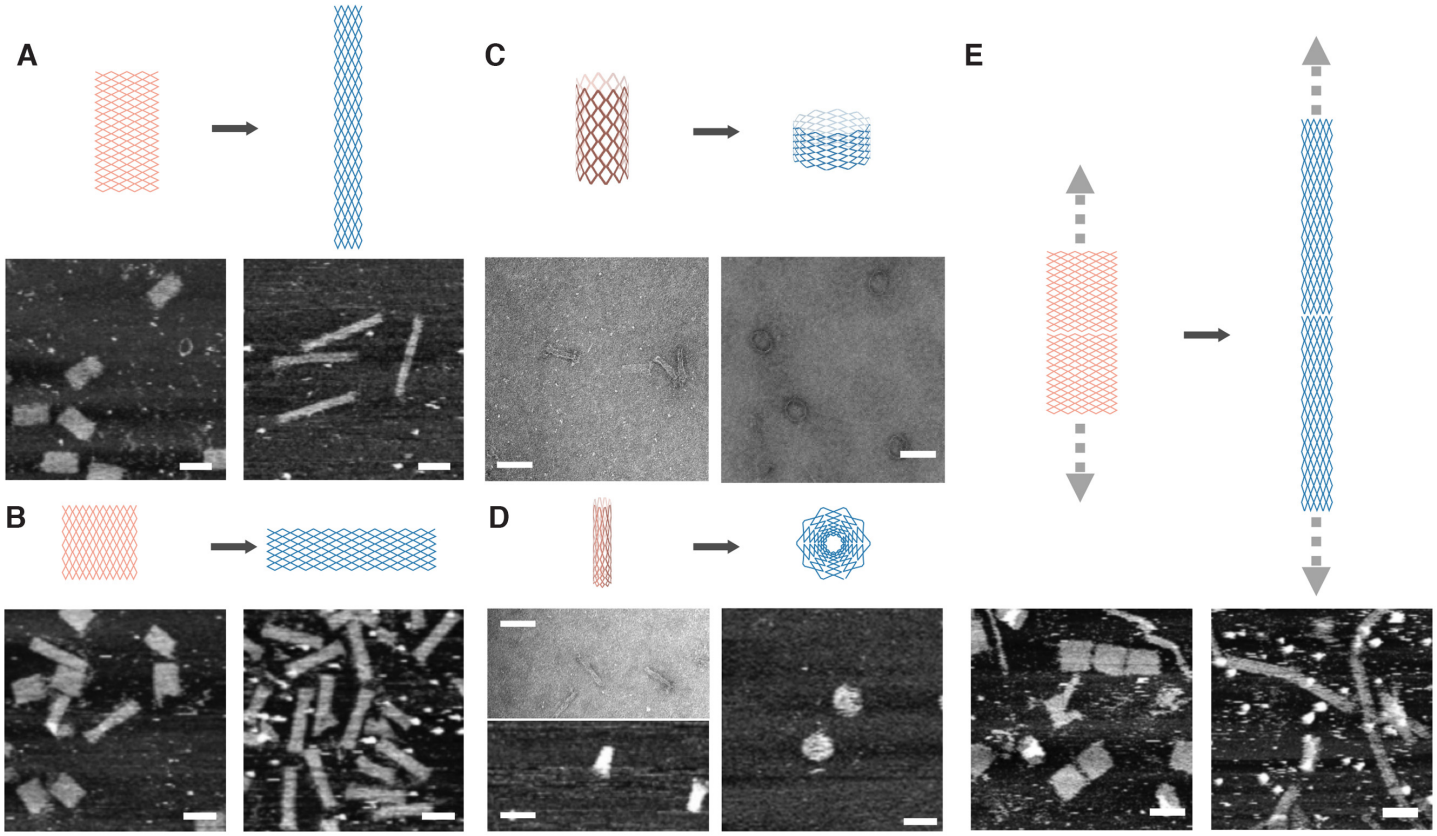
Allosteric transition of different DNA nanostructures based on DNA polymerase treatment. (**A**) Allosteric transition from an 8*B* × 31*H* rectangle to an 8*H* × 31*B* rectangle. (**B**) Allosteric transition from a 22*H* × 11*B* rectangle to a 22*B* × 11*H* rectangle. (**C**) Allosteric transition from a thin tube to a fat tube. (**D**) Allosteric transition from a tube to a disc. (**E**) Allosteric transition from a thick ribbon to a thin ribbon. Top: transition diagrams; bottom: AFM and TEM results (scale bars: 100 nm). Measurements are provided in Supplementary Tables S1 and S2.

Similar allosteric transition based on DNA polymerase treatment was also demonstrated in some other DNA nanostructures, and the corresponding allosteric states were characterized by AFM and TEM. Two more rectangles of different aspect ratios were tested. An 8*B* × 31*H* (measured at 67 ± 10 nm × 125 ± 10 nm, *N* = 30) rectangle turned to an 8*H* × 31*B* rectangle (measured at 30 ± 6 nm × 260 ± 23 nm, *N* = 30) (Figure 2A; Supplementary Figures S7 and S8), and a 22*H* × 11*B* rectangle (measured at 74 ± 8 nm × 79 ± 9 nm, *N* = 30) turned to a 22*B* × 11*H* rectangle (measured at 41 ± 6 nm × 180 ± 17 nm, *N* = 30) (Figure 2B; Supplementary Figures S9 and S10). Enzymatic gap filling also directed the transition from a thin tube of 22*H* (circumference) × 11*B* (height) to a fat tube of 22*B* (circumference) × 11*H* (height) (Figure 2C; Supplementary Figures S11 and S12). When another carefully designed tube was subjected to polymerase treatment, a disc (13 rings of helices of increasing lengths progressing away from the center) resulted (Figure 2D; Supplementary Figures S13 and S14). A multimer 10*B* × 24*H* rectangle took a thick ribbon (10*B*) shape initially (width measured at 97 ± 10 nm, *N* = 30), and it turned into thin ribbon (10*H*) after polymerase treatment (width measured at 31 ± 5 nm, *N* = 30) (Figure 2E; Supplementary Figures S15 and S16).

**Figure 3.**
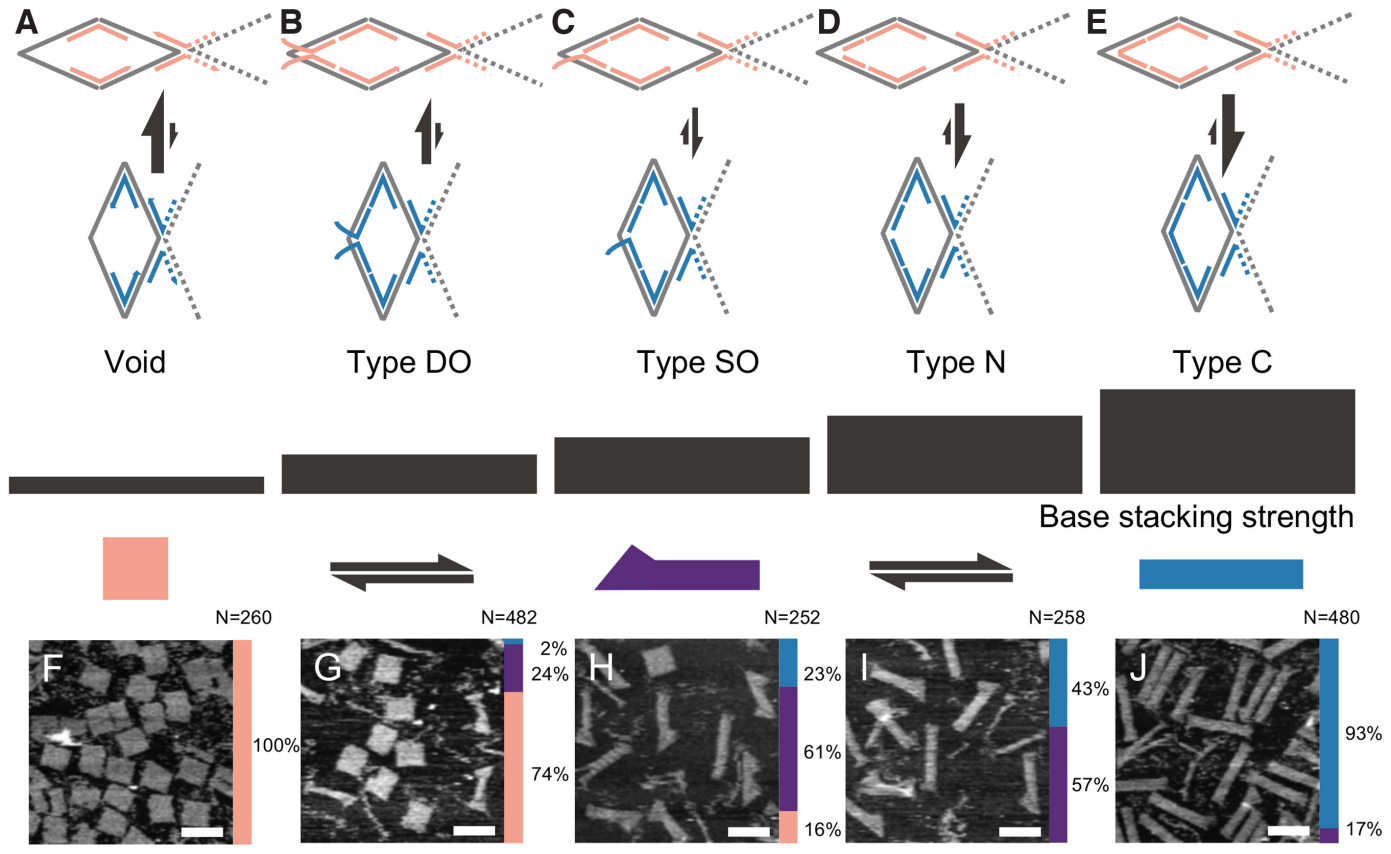
Ranking of stacking strengths for different types of effector staples. The stacking strengths are ranked from weakest to strongest: (**A**) void allosteric sites; (**B**) type DO effector staples; (**C**) type SO effector staples; (**D**) type N effector staples; and (**E**) type C effector staples. The incremental stacking strengths (as indicated by the arrow sizes of the local conformation equilibriums) of different types of effector staples are based on the transitional tendencies from a fat rectangle to a thin rectangle shown in AFM results (**F**–**J**). Allosteric state distributions (fat rectangle in coral, broom in purple and thin rectangle in blue) are shown in bar charts on the right-hand side of AFM images (details and statistics are provided in Supplementary Figure S18 and Supplementary Table S3). Scale bars: 150 nm.

Besides common continuous effector staples (type C) at allosteric sites, three other types of effector staples were also applied to fill the void allosteric sites (Figure 3A–E). When each type C effector staple was split in halves, a nick was available in the middle and we defined the split type as nicked effector staples (type N); single-stranded overhang(s) could be appended at the nick, and we defined the extended type at junction point as effector staples with single-branched overhangs (type SO) and effector staples with double-branched overhangs (type DO) (Supplementary Figure S17).

All four types of effector staples exhibited certain stacking strengths. According to the conventional wisdom, stacking strength of junctions with extended overhang(s) was believed to be limited or ignorable. In our systematic investigation, however, the stacking strength of such an overhang arrangement (types SO and DO) was still significant. We carefully investigated stacking strength of each type by the tendency of the allosteric transition from fat rectangle (10*B* × 25*H*) into thin rectangle (10*H* × 25*B*). According to the analysis of the AFM images, allosteric state distributions of different effector staple types were presented in bar charts, respectively, and a detailed comparison among different types was drawn (Figure 3, F–J, right; Supplementary Figure S18 and Supplementary Table S3). The population majority shift from fat rectangle to thin rectangle clearly demonstrated the incremental stacking strengths of the corresponding effector staples. With the void allosteric sites serving as a baseline (Figure 3A), the ranking of the stacking strength from the weakest to the strongest was as follows: type DO (Figure 3B), type SO (Figure 3C), type N (Figure 3D), and type C (Figure 3E) effector staples. Our energetic calculations revealed a similar trend (Supplementary Figure S19).

**Figure 4.**
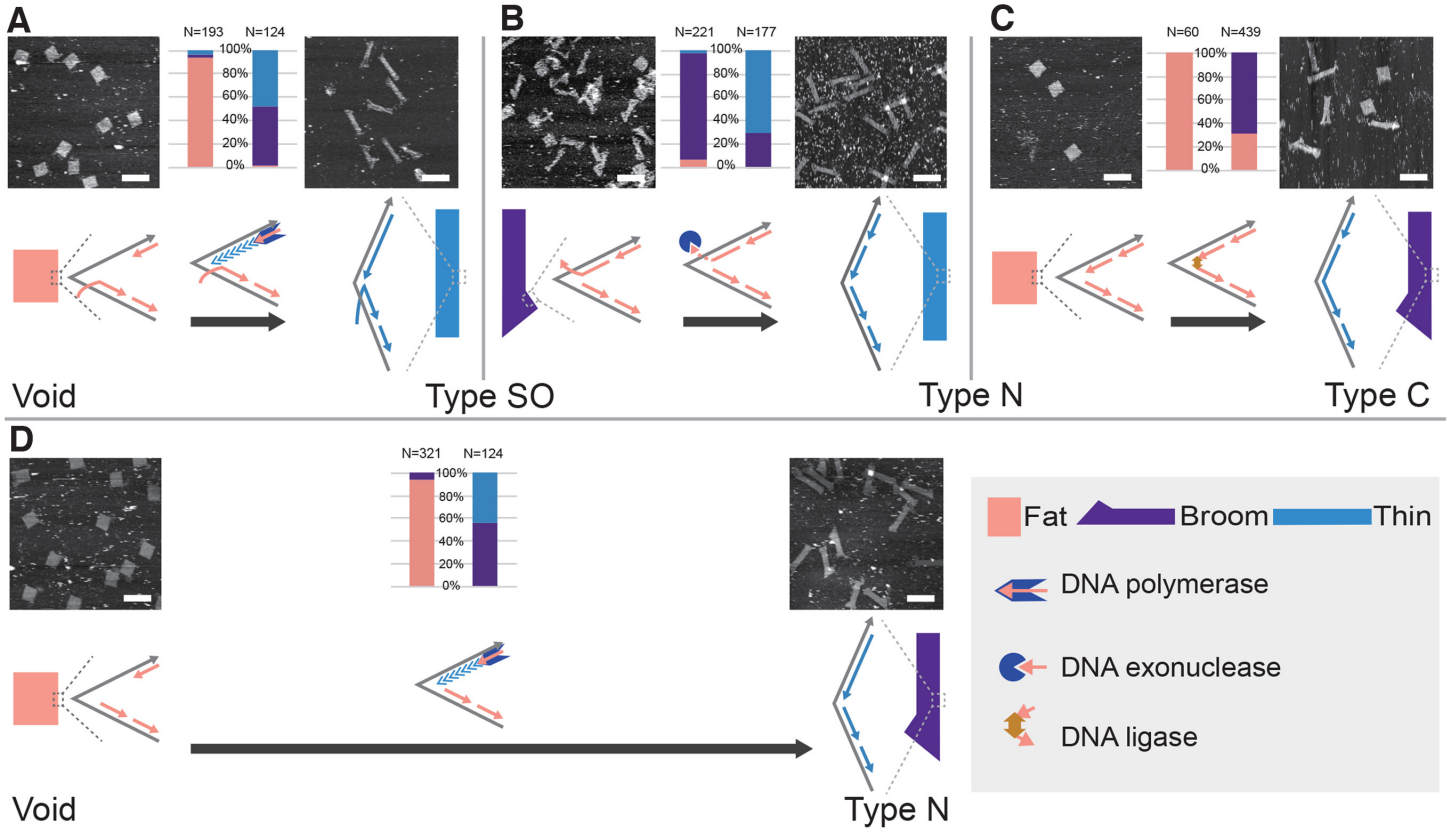
Enzymatic modification map. Based on the ranking of stacking strengths of different effector staples, the enzymatic modifications on effector staples result in a designated allosteric transition from a fat rectangle to a thin rectangle. The modifications of effector staples include (**A**) gap filling from void allosteric sites to type SO effector staples by DNA polymerase, (**B**) digestion from type SO effector staples to type N effector staples by exonuclease, (**C**) nick sealing from type N effector staples to type C effector staples by ligase, and (**D**) gap filling from void allosteric sites to type N effector staples by DNA polymerase. Bar charts of allosteric state distributions are shown by the sides of AFM image pairs for each kind of enzymatic modification (fat rectangle in coral, broom in purple and thin rectangle in blue). Schematics of effector staples with and without enzymatic modifications are placed close to the AFM images of the corresponding states. Scale bars: 200 nm. Details and statistics are provided in Supplementary Figures S20–S27 and Supplementary Table S4.

According to the ranking of stacking strengths, enzymes from a molecular cloning toolbox were applied to modify effector staples from one type to another for an enhanced stacking strength. The same DNA origami rectangle for stacking strength investigation was adopted in this series of examples. What we demonstrated next was the modification from type SO effector staples to type N effector staples by exonuclease. We tried a number of exonucleases but none of them showed desired digestion of the single-stranded overhangs and no notable allosteric transition was observed (results not shown). It was presumably due to the difficulty to obtain perfect digestion by exonucleases. Either an insufficient digestion or an overdigestion would not bring in stacking strength necessary to initiate an allosteric transition. Noting that many polymerases also have 3′–5′ exonuclease activity for the proofreading function, we applied Q5 DNA polymerase to trim the overhangs of the effector staples. To our surprise, the exonuclease activity of Q5 polymerase turned the type SO effector staples to the type N effector staples and the desired allosteric transition was achieved (Figure 4B; Supplementary Figures S20 and S21).

We then sought to modify type N effector staples to type C and T4 ligase was applied for such a task. Because of the substantial stacking strength, we only placed 8 of 24 pairs of type N effector staples for the 10*B* × 25*H* origami structure to maintain a fat rectangle conformation initially. Broom-like structures resulted after the ligation treatment, indicating deficient transition from a limited number of type C effector staples (Figure 4C; Supplementary Figures S22–S24).

From different initial staple placements on allosteric sites, the gap filling can result in not only the type C effector staples (Supplementary Figure S27) but also type SO (Figure 4A; Supplementary Figure S25) and type N (Figure 4D; Supplementary Figure S26) effector staples for an allosteric transition. In all these three examples, significant allosteric transitions from fat rectangles to thin rectangles were presented under AFM.

## DISCUSSION

We have already shown in an earlier study that the controllable allosteric transitions by hybridizing effector staples are reversible (15). The exclusion of effector staples can turn allosteric transition back to the original state. The enzymatic modification is potentially reversible as well, but the challenge is to discriminate the modified effector staples from other common staples. For example, if allosteric sites of a custom sequenced scaffold are prespecified as restriction sites, specific nicking endonucleases can be introduced to treat effector staples, which serves as the reverse reaction to ligation. The sequence-specific nicking enzymatic treatment can also be introduced by guide RNA/DNA-based enzymes such as Cas9 and artificial restriction enzymes (18–20). Another way to introduce discrimination is to apply different species of nucleic acids or modified bases to effector staples, and reversible transition can be realized by specific treatment on the chosen species of nucleic acids.

The controllable allostery also suggests that reconfigurable structures can provide an attractive single-molecule platform to study not just enzymatic activity but also binding between nucleic acids and proteins quantitatively (21,22). When a working interface between nucleic acids and the associated enzymes or proteins is well integrated in the synthetic allosteric system, it can become a reliable reporter of enzymatic activities and protein–DNA interactions. Moreover, one can also imagine to engineer the allosteric system *in vivo*, so that a certain biomolecular interaction or process can be reported and amplified by the conformational readout (23).

## Supplementary Material

gkaa488_Supplemental_Files

## References

[1] Nussinov R., Tsai C.-J. Allostery in disease and in drug discovery. Cell. 2013; 153:293–305. 23582321 10.1016/j.cell.2013.03.034

[2] Motlagh H.N., Wrabl J.O., Li J., Hilser V.J. The ensemble nature of allostery. Nature. 2014; 508:331–339. 24740064 10.1038/nature13001 PMC4224315

[3] Nussinov R., Tsai C.-J., Liu J. Principles of allosteric interactions in cell signaling. J. Am. Chem. Soc. 2014; 136:17692–17701. 25474128 10.1021/ja510028c PMC4291754

[4] Kim S., Broströmer E., Xing D., Jin J., Chong S., Ge H., Wang S., Gu C., Yang L., Gao Y.Q. Probing allostery through DNA. Science. 2013; 339:816–819. 23413354 10.1126/science.1229223 PMC3586787

[5] Yan L., Chen Z. A unifying mechanism of DNA translocation underlying chromatin remodeling. Trends Biochem. Sci. 2019; 45:217–227. 31623923 10.1016/j.tibs.2019.09.002

[6] Rothemund P.W. Folding DNA to create nanoscale shapes and patterns. Nature. 2006; 440:297–302. 16541064 10.1038/nature04586

[7] Dietz H., Douglas S.M., Shih W.M. Folding DNA into twisted and curved nanoscale shapes. Science. 2009; 325:725–730. 19661424 10.1126/science.1174251 PMC2737683

[8] Douglas S.M., Dietz H., Liedl T., Högberg B., Graf F., Shih W.M. Self-assembly of DNA into nanoscale three-dimensional shapes. Nature. 2009; 459:414–418. 19458720 10.1038/nature08016 PMC2688462

[9] Castro C.E., Kilchherr F., Kim D.-N., Shiao E.L., Wauer T., Wortmann P., Bathe M., Dietz H. A primer to scaffolded DNA origami. Nat. Methods. 2011; 8:221–229. 21358626 10.1038/nmeth.1570

[10] Han D., Pal S., Nangreave J., Deng Z., Liu Y., Yan H. DNA origami with complex curvatures in three-dimensional space. Science. 2011; 332:342–346. 21493857 10.1126/science.1202998

[11] Benson E., Mohammed A., Gardell J., Masich S., Czeizler E., Orponen P., Högberg B. DNA rendering of polyhedral meshes at the nanoscale. Nature. 2015; 523:441–444. 26201596 10.1038/nature14586

[12] Gerling T., Wagenbauer K.F., Neuner A.M., Dietz H. Dynamic DNA devices and assemblies formed by shape-complementary, non–base pairing 3D components. Science. 2015; 347:1446–1452. 25814577 10.1126/science.aaa5372

[13] Shrestha P., Emura T., Koirala D., Cui Y., Hidaka K., Maximuck W.J., Endo M., Sugiyama H., Mao H. Mechanical properties of DNA origami nanoassemblies are determined by Holliday junction mechanophores. Nucleic Acids Res. 2016; 44:6574–6582. 27387283 10.1093/nar/gkw610 PMC5001620

[14] Song J., Li Z., Wang P., Meyer T., Mao C., Ke Y. Reconfiguration of DNA molecular arrays driven by information relay. Science. 2017; 357:eaan3377. 28642234 10.1126/science.aan3377

[15] Cui Y., Chen R., Kai M., Wang Y., Mi Y., Wei B. Versatile DNA origami nanostructures in simplified and modular designing framework. ACS Nano. 2017; 11:8199–8206. 28654269 10.1021/acsnano.7b03187

[16] Gerling T., Dietz H. Reversible covalent stabilization of stacking contacts in DNA assemblies. Angew. Chem. 2019; 131:2706–2710. 10.1002/anie.201812463 PMC6984961 30694591

[17] Agarwal N.P., Matthies M., Joffroy B., Schmidt T.L. Structural transformation of wireframe DNA origami via DNA polymerase assisted gap-filling. ACS Nano. 2018; 12:2546–2553. 29451771 10.1021/acsnano.7b08345

[18] Enghiad B., Zhao H. Programmable DNA-guided artificial restriction enzymes. ACS Synth. Biol. 2017; 6:752–757. 28165224 10.1021/acssynbio.6b00324

[19] Jinek M., Chylinski K., Fonfara I., Hauer M., Doudna J.A., Charpentier E. A programmable dual-RNA–guided DNA endonuclease in adaptive bacterial immunity. Science. 2012; 337:816–821. 22745249 10.1126/science.1225829 PMC6286148

[20] Gasiunas G., Barrangou R., Horvath P., Siksnys V. Cas9–crRNA ribonucleoprotein complex mediates specific DNA cleavage for adaptive immunity in bacteria. Proc. Natl Acad. Sci. U.S.A. 2012; 109:15539–15540. 10.1073/pnas.1208507109 PMC3465414 22949671

[21] Nickels P.C., Wunsch B., Holzmeister P., Bae W., Kneer L.M., Grohmann D., Tinnefeld P., Liedl T. Molecular force spectroscopy with a DNA origami–based nanoscopic force clamp. Science. 2016; 354:305–307. 27846560 10.1126/science.aah5974 PMC6546592

[22] Funke J.J., Ketterer P., Lieleg C., Schunter S., Korber P., Dietz H. Uncovering the forces between nucleosomes using DNA origami. Sci. Adv. 2016; 2:e1600974. 28138524 10.1126/sciadv.1600974 PMC5262459

[23] Delebecque C.J., Lindner A.B., Silver P.A., Aldaye F.A. Organization of intracellular reactions with rationally designed RNA assemblies. Science. 2011; 333:470–474. 21700839 10.1126/science.1206938

