# Supplementary material for: Allostery of DNA nanostructures controlled by enzymatic modifications
Source: Nucleic Acids Res. 2020 Jun 11;48(13):7595–600. doi: 10.1093/nar/gkaa488 (PMC7367186; doi:10.1093/nar/gkaa488)
Supplement: gkaa488_Supplemental_Files [file gkaa488_supplemental_files.zip › SI.pdf]

Supplementary Information

Allostery of DNA nanostructures controlled by enzymatic modifications

Qi Yan[†], Yaqi Wang[†], Jile Shi, Bryan Wei[*]

School of Life Sciences, Tsinghua University-Peking University Center for Life Sciences, Center for Synthetic and Systems Biology, Tsinghua University, Beijing 100084, China

[†]These authors contributed equally.

[*]Correspondence and requests for materials should be addressed to B.W. (email: bw@tsinghua.edu.cn)

Table of Contents

1. Results of allostery from DNA polymerase-based gap filling........................3

1.1 Allostery based on strand hybridization 3

1.2 Allostery of different DNA nanostructures with DNA polymerase treatment 4

2. Results of stacking strength with different types of effector staples...........21

2.1 Stacking strength gradient at allosteric sites with different types of effector staples 21

2.2 Sub-classification of different types of effector staples 22

2.3 Calculation of free energies at allosteric sites with different types of effector staples 24

3. Results of allostery transition map with different enzyme treatments.......25

3.1 Allosteric transition with exonuclease 25

3.2. Allosteric transition with ligase...27

3.3. Allosteric transition with polymerase...30

3.4 Allosteric states distribution without and with enzyme treatment 33

1. Results of allostery from DNA polymerase-based gap filling

1.1 Allostery based on strand hybridization

Figure S1 shows the structure design details and the corresponding allosteric transition based on strand hybridization, which serves as a positive control of allosteric transition based on enzymatic elongation.

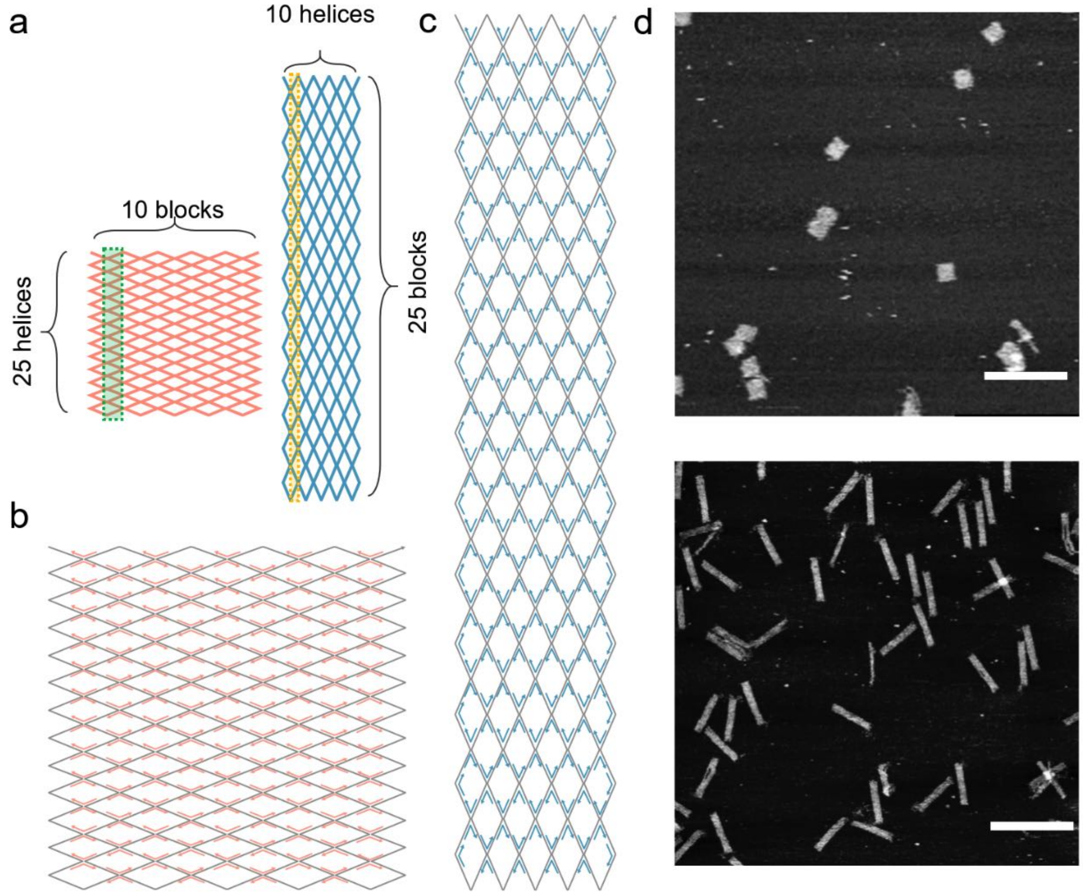

Figure S1. Allosteric transition from a 25H×10B rectangle to a 25B×10H rectangle based on strand hybridization. a. Schematic diagrams of DNA nanostructures before and after allosteric transition, in which a block (in the green box) and a helix (in the yellow box) are highlighted. Schematic diagrams of conformations with strand level of details are provided in b (25H×10B rectangle) and c (25B×10H rectangle). d. Corresponding AFM results of 25H×10B rectangle (top) and 25B×10H rectangle (bottom). Effector staples were hybridized to the preformed structure in a annealing program from 60 °C to 25 °C for 14 h before imaging. Scale bars: 400 nm.

1.2 Allostery of different DNA nanostructures with DNA polymerase treatment

Figure S2-S16 show the details of structure designs and DNA polymerase gap filling, as well as the full-size AFM figures. Tables S1 and S2 show the measurements of DNA nanostructures based on AFM and TEM results.

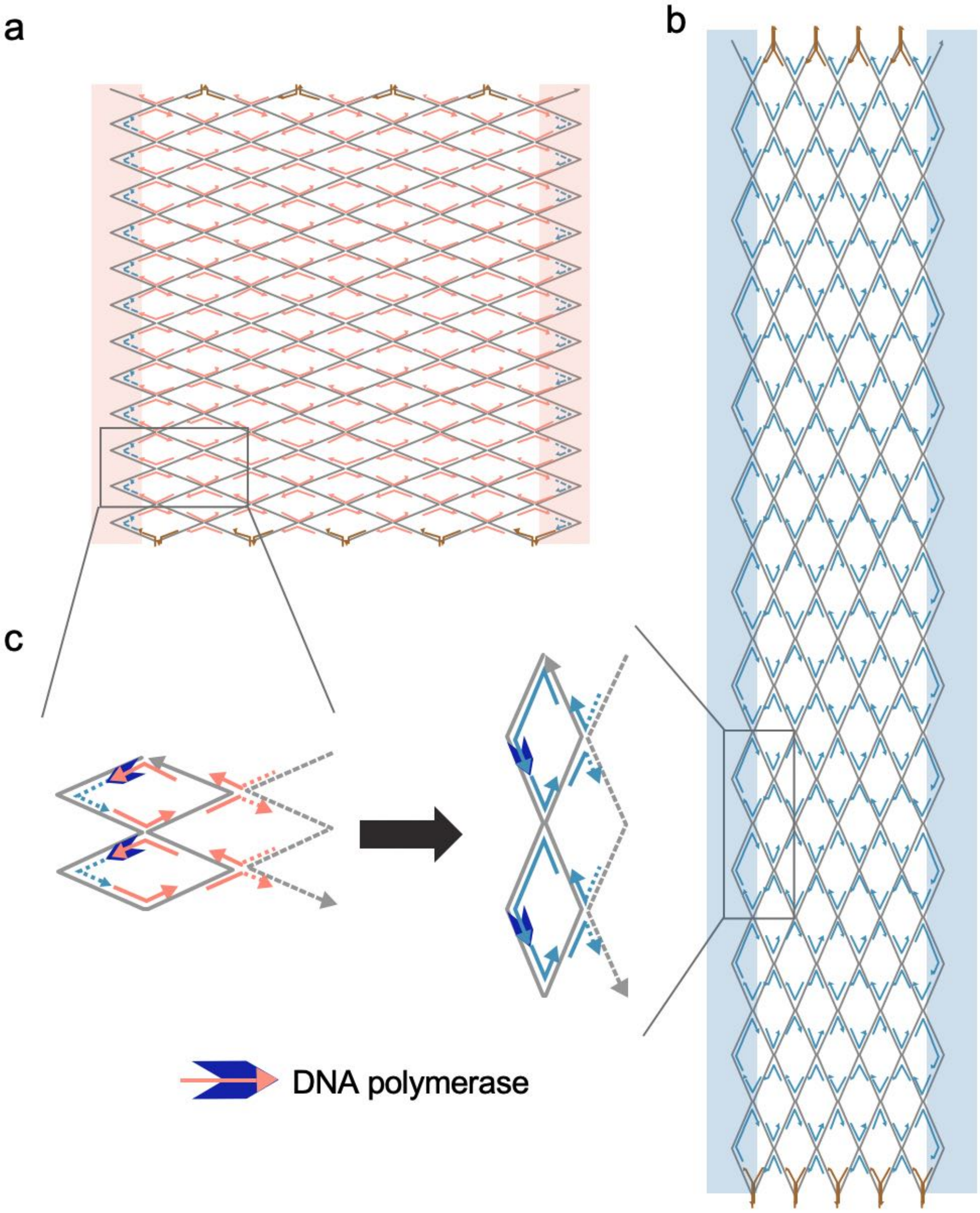

Figure S2. Allosteric transition from a 25H×10B rectangle to a 25B×10H rectangle based on DNA polymerase elongation. Schematic diagrams of conformations with strand level of details are provided in a (25H×10B rectangle) and b (25B×10H rectangle). The allosteric sites are highlighted. The brown staples on both top and bottom of the DNA nanostructure are designed for two purposes: weakening stacking strength and blocking non-specific elongation of DNA polymerase. c. Working model of allosteric transition based on DNA polymerase treatment.

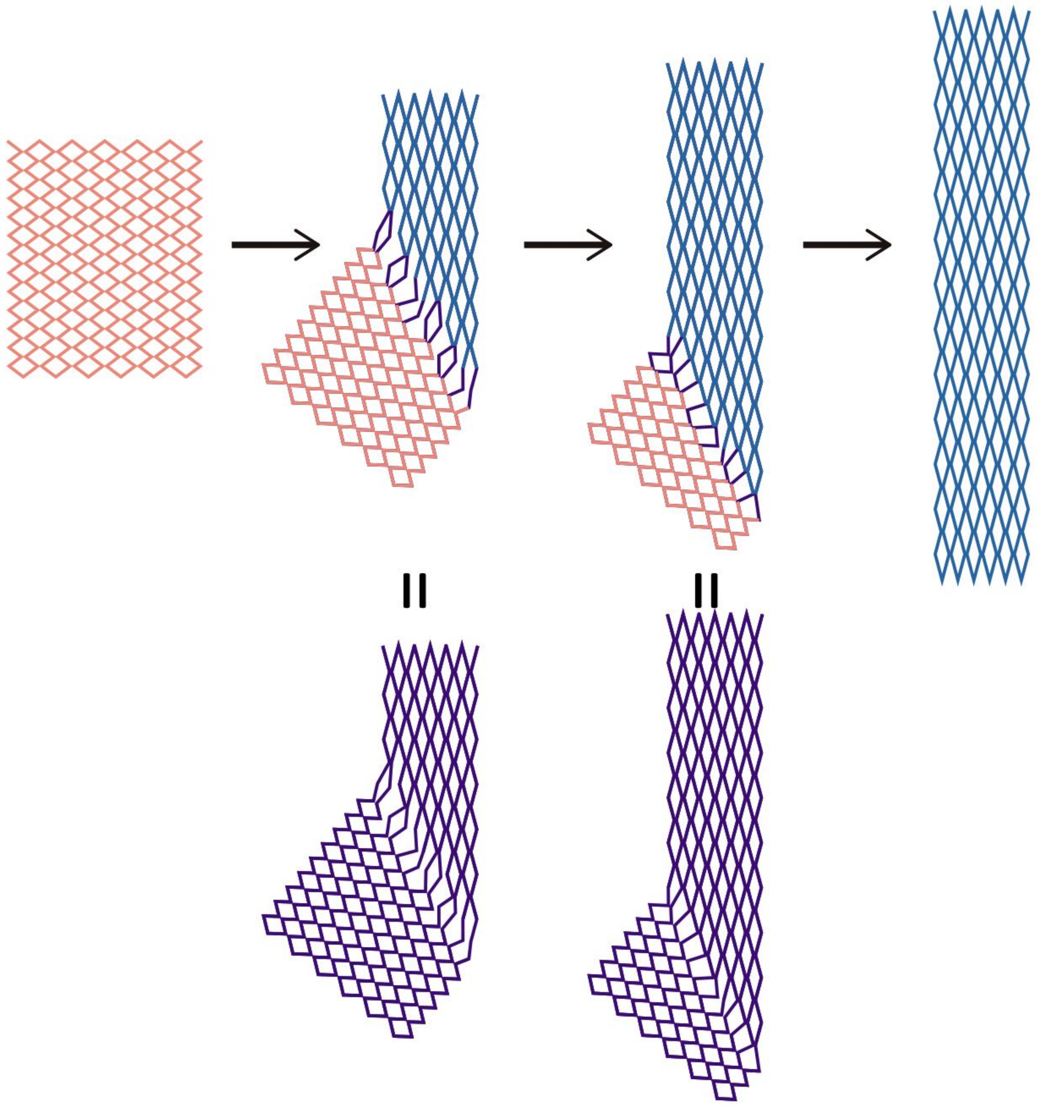

Figure S3. Gradual conformation transion. The dynamic process is initiated from the allosteric sites (as pointed out by arrows). Conformation of fat rectangle is shown in coral. Conformation of thin rectangle is shown in blue. Intermediate state of broom-like configuration is also shown in purple to match the general color scheme.

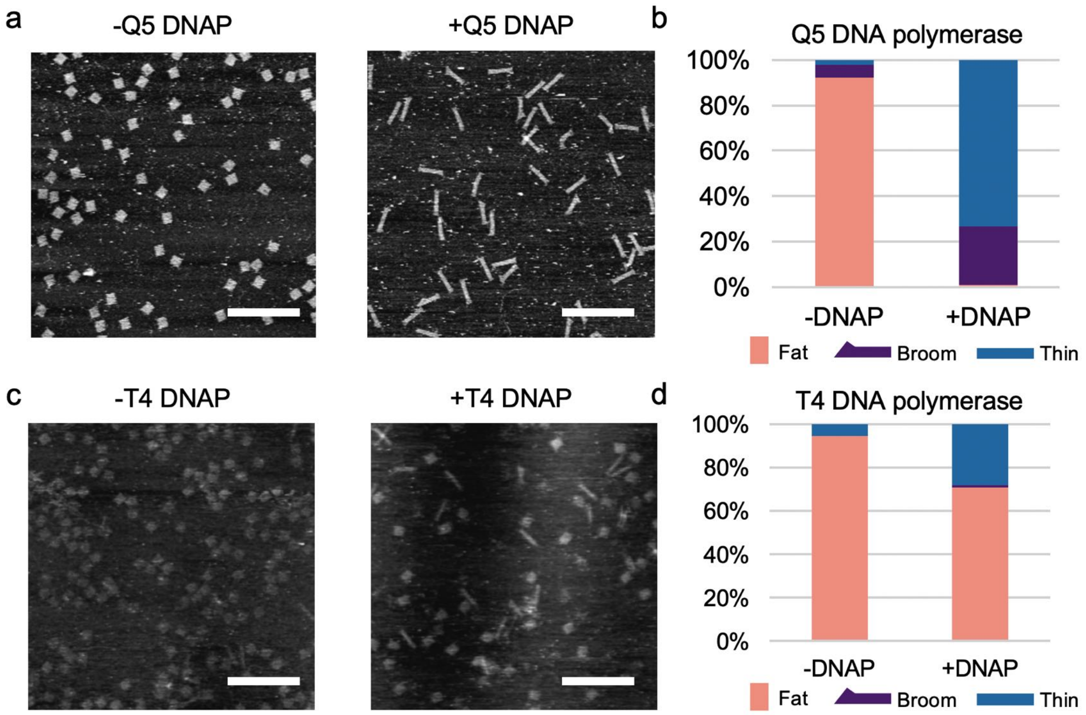

Figure S4. Allostery transition with different DNA polymerases. a. AFM results of the allosteric transition with Q5 DNA polymerase treatment. Samples with and without Q5 DNA polymerase were incubated at 37 °C for 5 h before imaging. Without DNA polymerase treatment, the formation yield is 97.1% (N=240). With DNA polymerase treatment, the formation yield is 99.4% (N=171). There is no apparent drop of yield after enzymatic treatment. b. Distributions of allosteric states without (left) and with (right) Q5 DNA polymerase treatment. c. AFM results of the allosteric transition with T4 DNA polymerase treatment. Samples with and without T4 DNA polymerase (in NEBuffer™ 2.1) were incubated at 25 °C for 17 h before imaging. d. Distributions of allosteric states without (left) and with (right) T4 DNA polymerase treatment. The results with limited optimization indicate a better gap filling ability for Q5 DNA polymerase compared to T4 DNA polymerase. Scale bars: 600 nm.

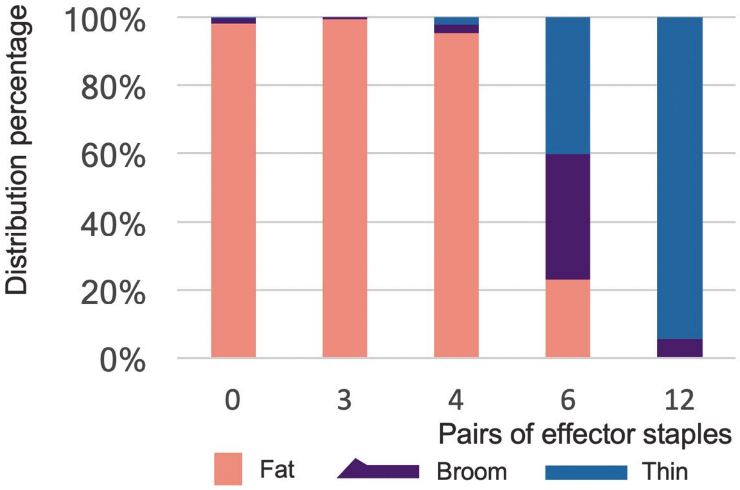

Figure S5. Distribution of allosteric states with different pairs of effector staples. Disturbution percentage of allosteric states when the pairs of type C effector staples are 0, 3, 4, 6 and 12 respectively. When the distribution of allosteric states with DNA polymerase treatment (Figure 1C) is fitted to this bar chart, the efficiency of the enzymatic elongation by Q5 DNA polymerase is between 50% (6 pairs) and 100% (12 pairs).

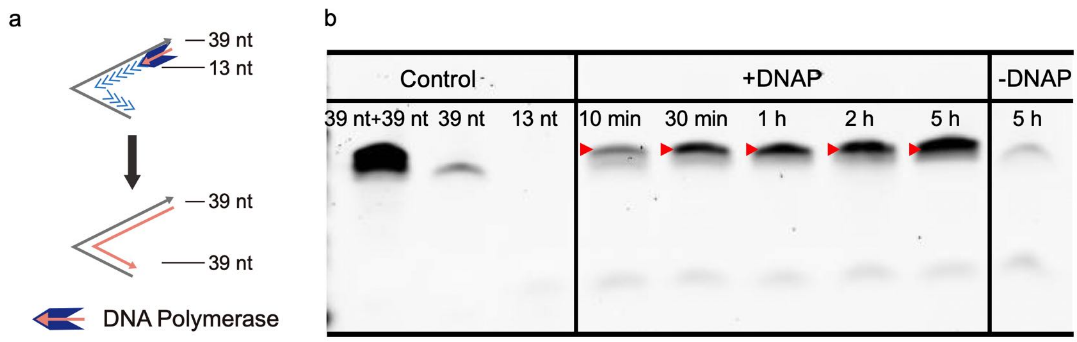

Figure S6. Verification of polymerase activity of Q5 DNA polymerase. a. Working model of DNA polymerase treatment. The lengths of each strand are specified. b. Polyacrylamide gel electrophoresis results of polymerase treatment. The increasing intensity of the 39-nt bands over the time course indicates an efficient elongation (from 13 nt to 39 nt by the polymerase activity of Q5 DNA polymerase). Samples with Q5 DNA polymerase were incubated at 37°C for 10 min, 30 min, 1 h, 2 h and 5 h before characterization, and sample without Q5 DNA polymerase was incubated at 37°C for 5 h before characterization. The solid triangles point at 39-nt bands.

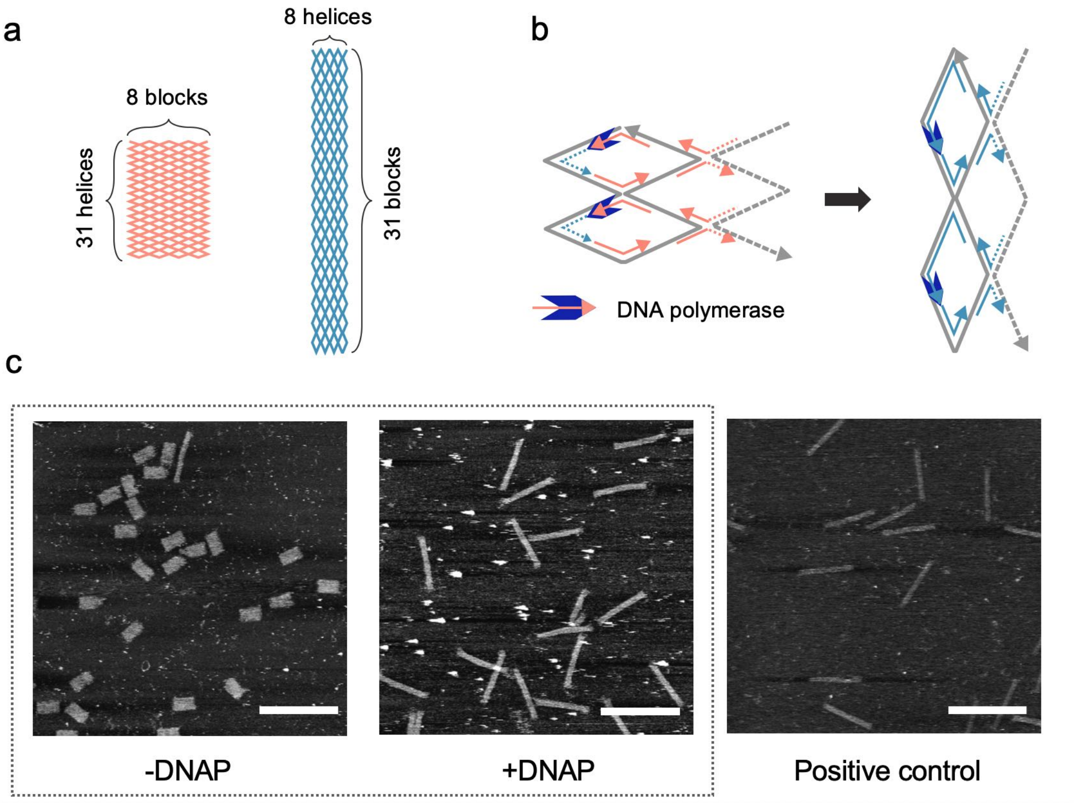

Figure S7. Allosteric transition from an 8B × 31H rectangle to an 8H× 31B rectangle based on DNA polymerase gap filling. a. Schematic diagrams of 8B×31H rectangle (left) and 8H×31B rectangle (right). b. Working model of allosteric transition based on DNA polymerase treatment. c. AFM results of the allosteric transition. Left: 8B×31H rectangle without enzyme treatment; middle: 8H×31B rectangle with enzyme treatment; right: 8H×31B rectangle with type C effector staples as a positive control. Samples with and without Q5 DNA polymerase were incubated at 37 °C for 5 h before imaging. Scale bars: 400 nm.

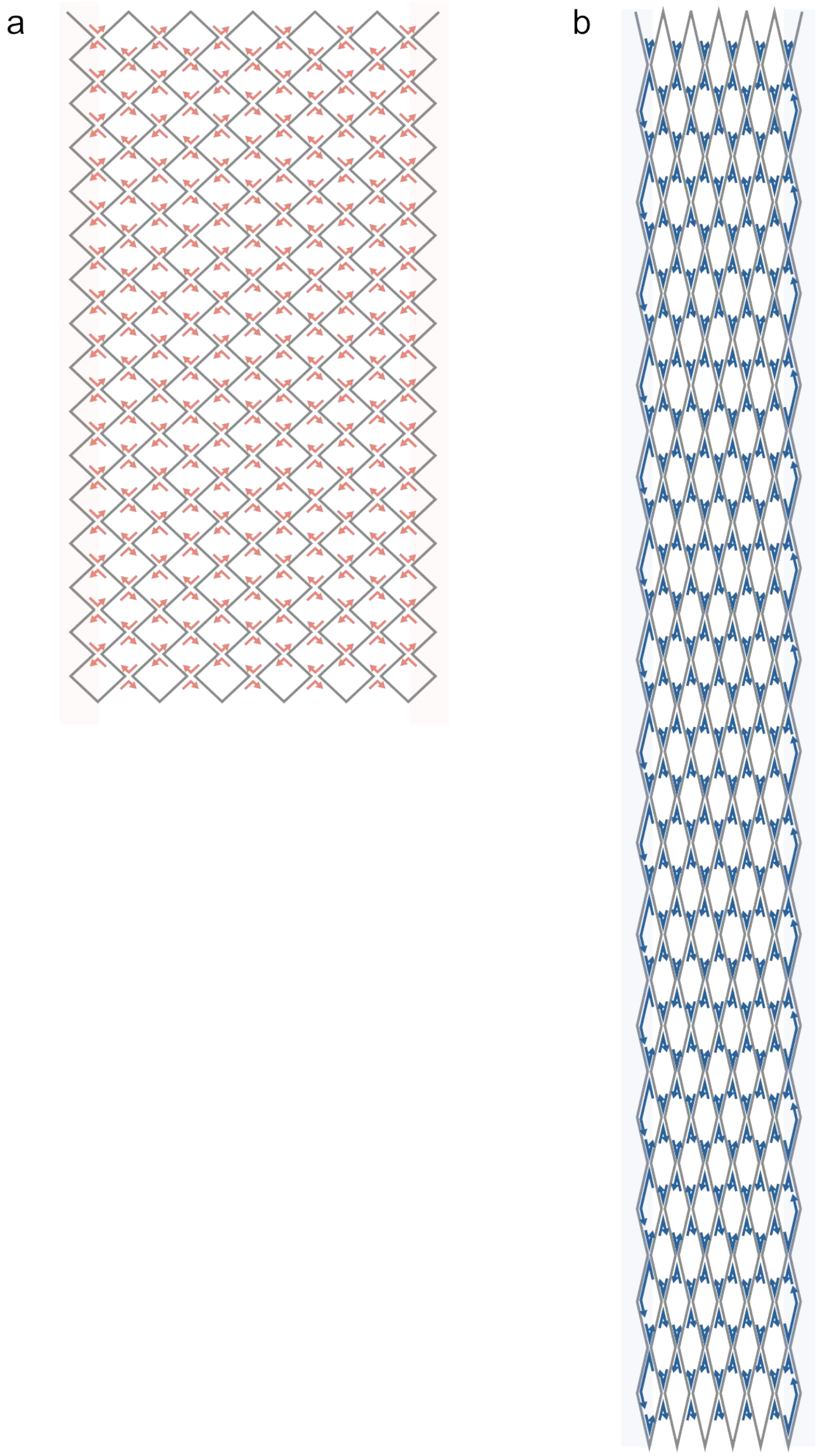

Figure S8. Schematic diagrams of conformations with strand level of details are provided in a (8B×31H rectangle) and b (8H×31B rectangle). The allosteric sites are highlighted.

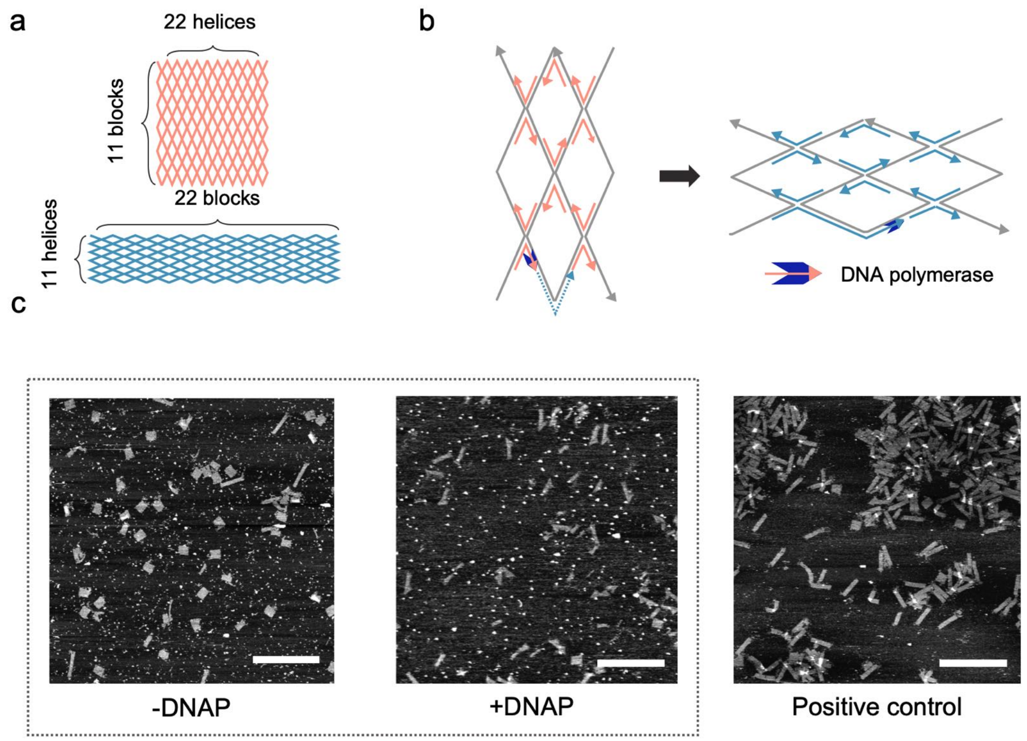

Figure S9. Allosteric transition from a 22H×11B rectangle to a 22B×11H rectangle based on DNA polymerase gap filling. a. Schematic diagrams of 22H×11B rectangle (left) and 22B×11H rectangle (right). b. Working model of allosteric transition based on DNA polymerase treatment. The local type C effector staples generated at allosteric sites cascade into the global structure allostery. c. AFM results of the allostery of 22B×11H rectangle. Left: 22H×11B rectangle without enzyme treatment; middle: 22H×11B rectangle with enzyme treatment; right: 22B×11H rectangle positive control with type C effector staples. Samples with and without Q5 DNA polymerase were incubated at 37 °C for 5 h before imaging. Scale bars: 600 nm.

a

b

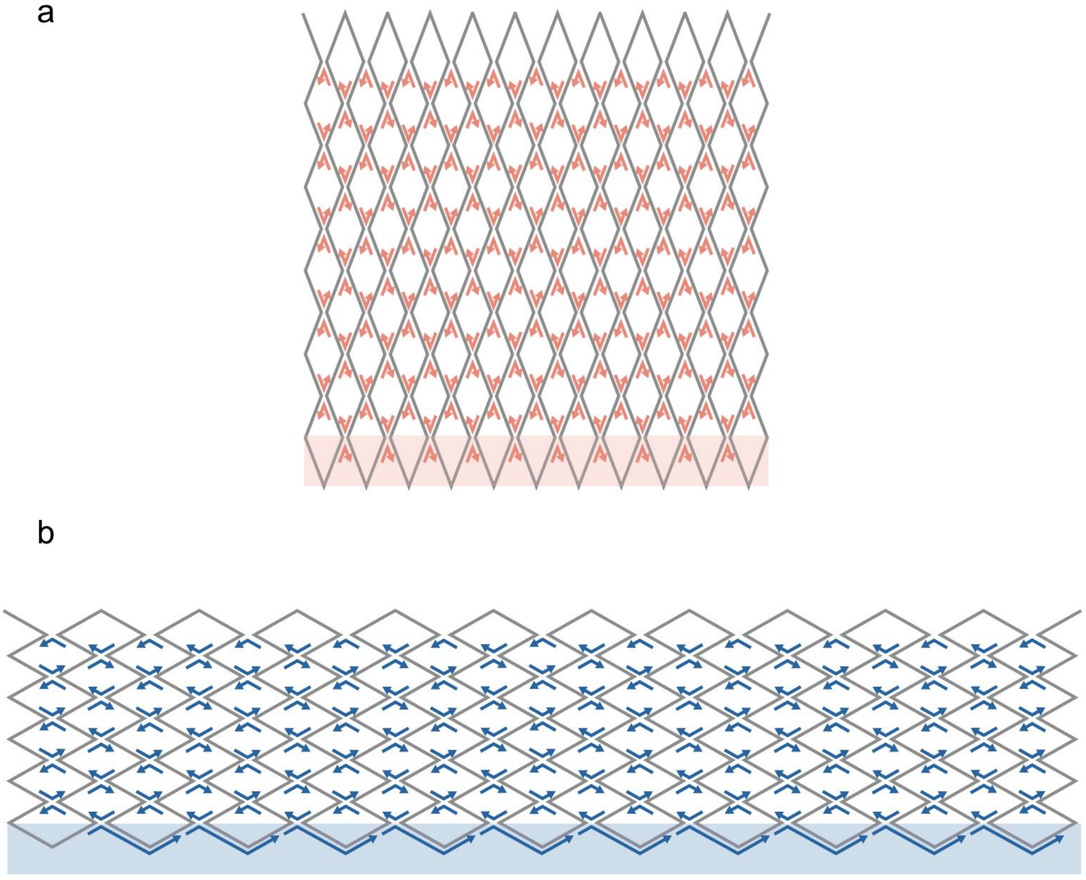

Figure S10. Schematic diagrams of conformations with strand level of details are provided in a (22H×11B rectangle) and b (22B×11H rectangle). The allosteric sites are highlighted. Effector staples are designed for the bottom boudary highlighted to avoid undesired allostery before enzymatic treatment.

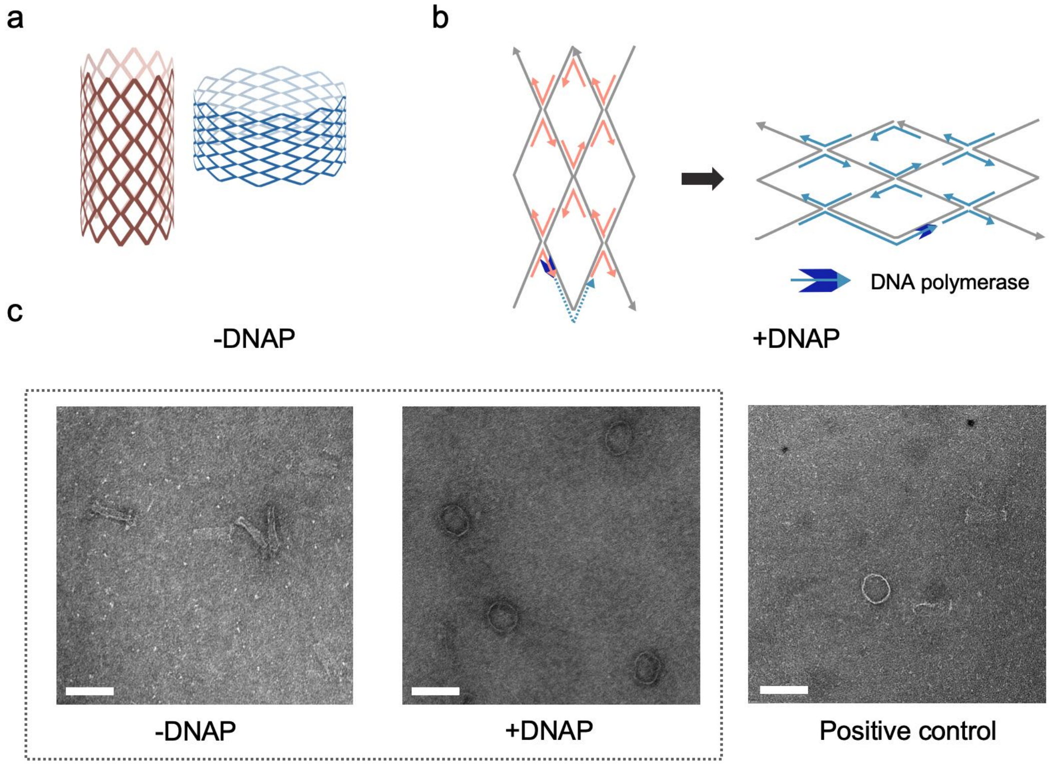

Figure S11. Allosteric transition from a 22H×11B thin tube to a 22B×11H fat tube based on DNA polymerase gap filling. a. Schematic diagrams of 22H×11B thin tube (left) and 22B×11H fat tube (right). b. Working model of allosteric transition based on DNA polymerase treatment. c. TEM results of the allostery transition from thin tube to fat tube. Left: 22H×11B tube without enzyme treatment; middle: 22H×11B tube with enzyme treatment; right: 22B×11H tube positive control with type C effector staples. Samples with and without Q5 DNA polymerase were incubated at 37 °C for 5 h before imaging. Scale bars: 600 nm.

a

b

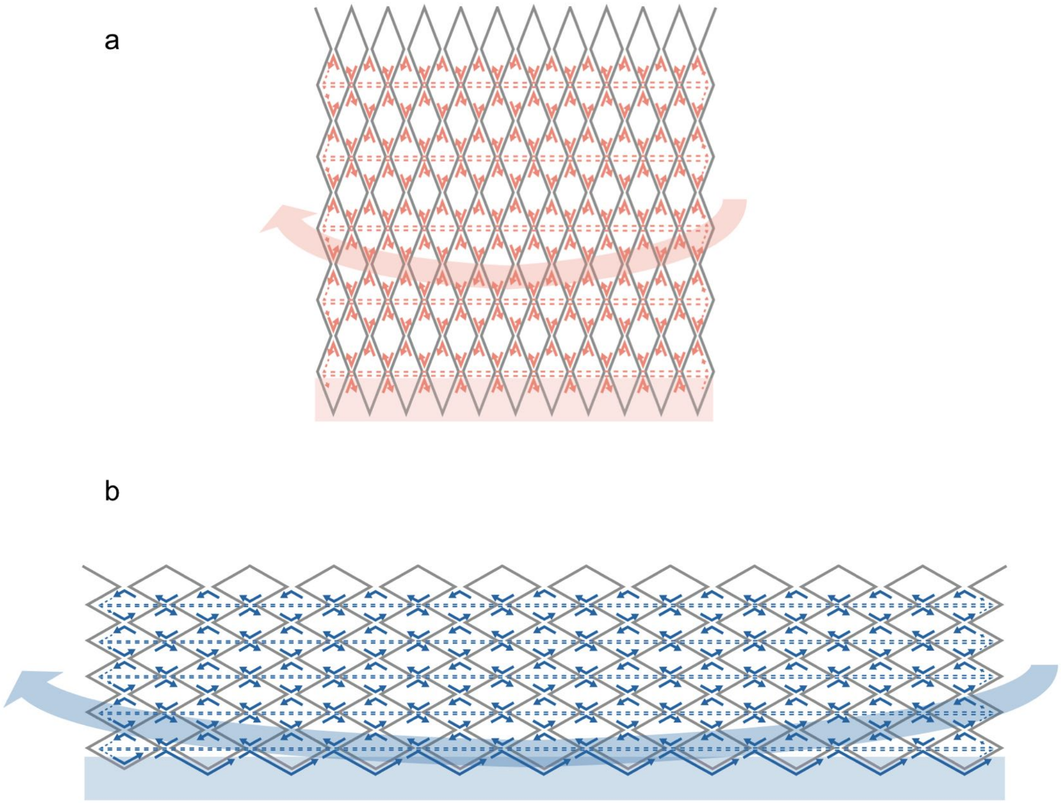

Figure S12. Schematic diagrams of conformations with strand level of details are provided in a (22H×11B thin tube) and b (22B×11H fat tube). The allosteric sites are highlighted. Effector staples are designed for the bottom boudary highlighted to avoid undesired allostery before enzymatic treatment.

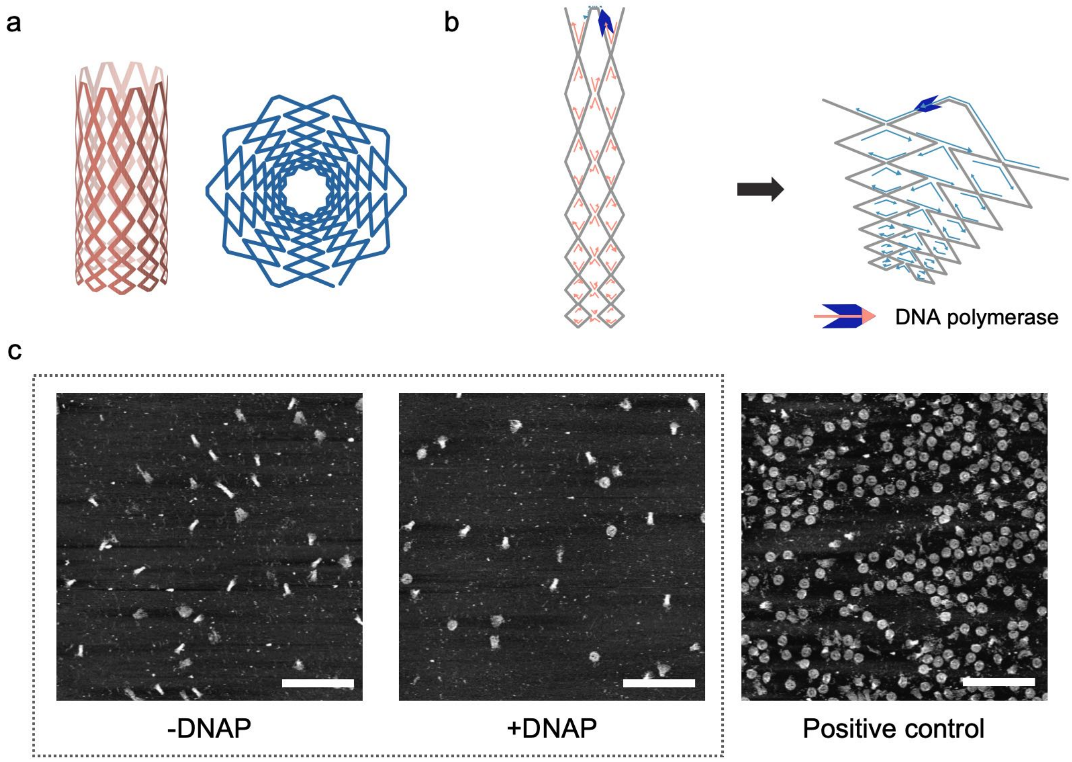

Figure S13. Allosteric transition from a 13H tube to a 13H disc based on DNA polymerase gap filling. a. Schematic diagrams of 20H (circumference)×13B (height) tube and 20B×13H disc. The block lengths are incremental from one end to the other end in tube conformation or from outermost ring to innermost ring in disc conformation. b. Working model of allosteric transition based on DNA polymerase treatment. c. AFM results of the experiment group and negative control. The left AFM picture corresponds to the negative control whereas the middle AFM picture corresponds to the experiment group. Left: 13H ring without enzyme treatment; middle: 13H ring with enzyme treatment; right:13H disc positive control with type C effector staples. Samples with and without Q5 DNA polymerase were incubated at 37 °C for 5 h before imaging. Scale bars: 600 nm.

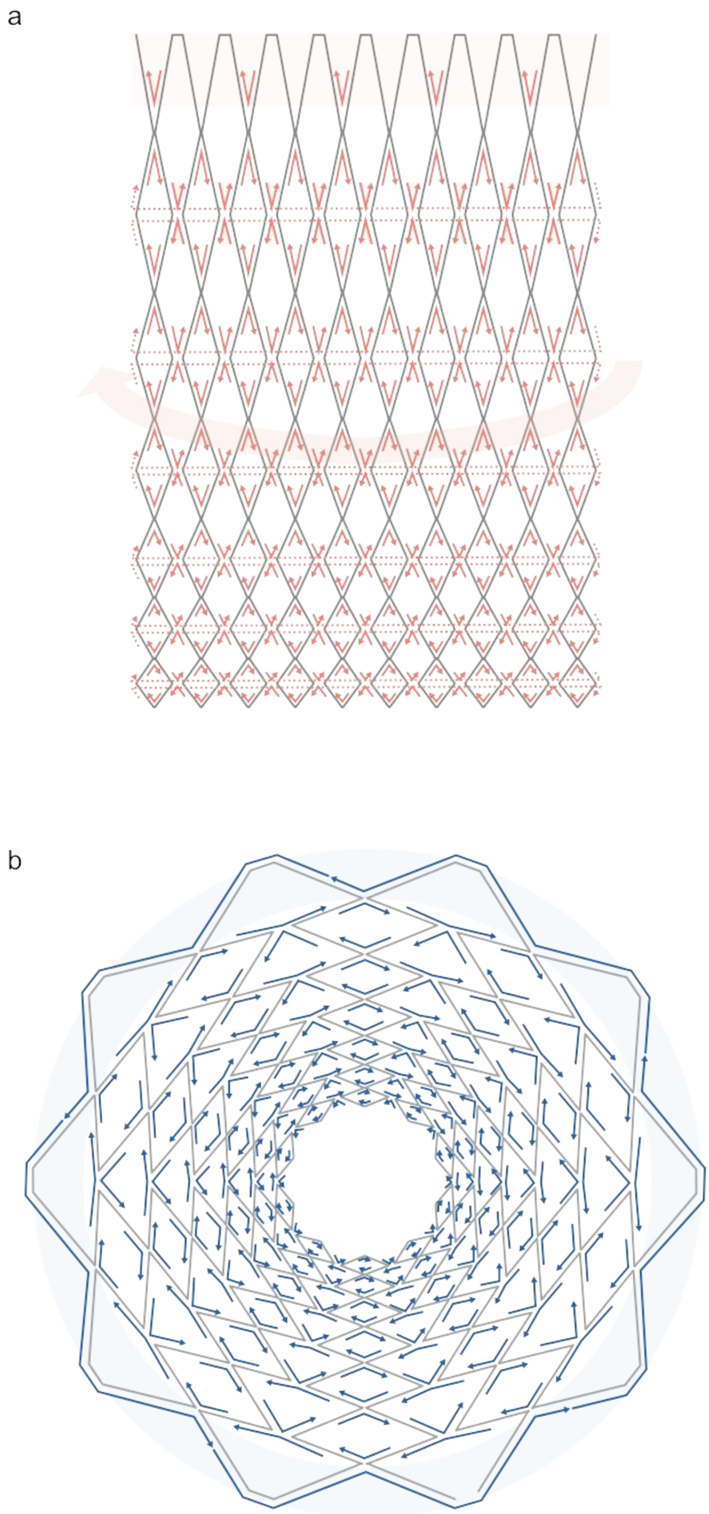

Figure S14. Schematic diagrams of conformations with strand level of details are provided in a (20H×13B tube) and b (20B×13H disc). The allosteric sites are highlighted. Effector staples are designed for the outer boudary highlighted to avoid undesired allostery before enzymatic treatment.

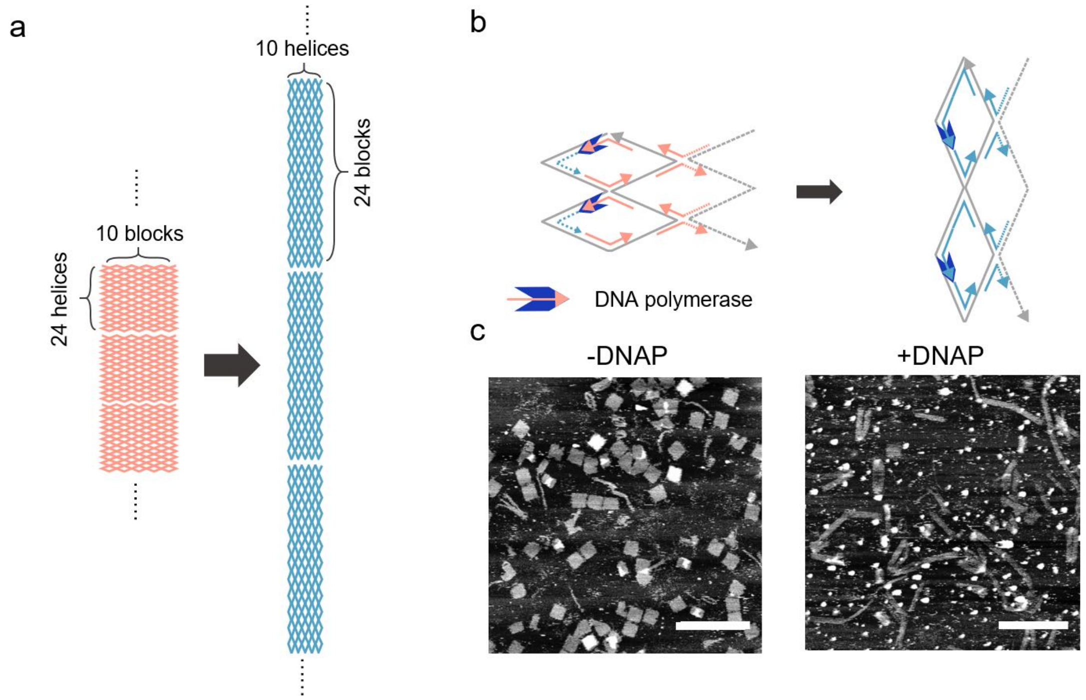

Figure S15. Allosteric transition from a 10B fat ribbon to a 10H thin ribbon based on DNA polymerase gap filling. a. Schematic diagrams of a 10B×24H rectangle multimer and a 10H×24B rectangle multimer. b. Working model of allosteric transition based on DNA polymerase treatment. c. AFM results of the allostery of 10B×24H rectangle polymer. Left: 10B fat ribbon without enzyme treatment; middle: 10B fat ribbon with enzyme treatment; right: 10H thin ribbon positive control with type C effector staples. Samples with and without Q5 DNA polymerase were incubated at 37 °C for 5 h before imaging. Scale bars: 600 nm.

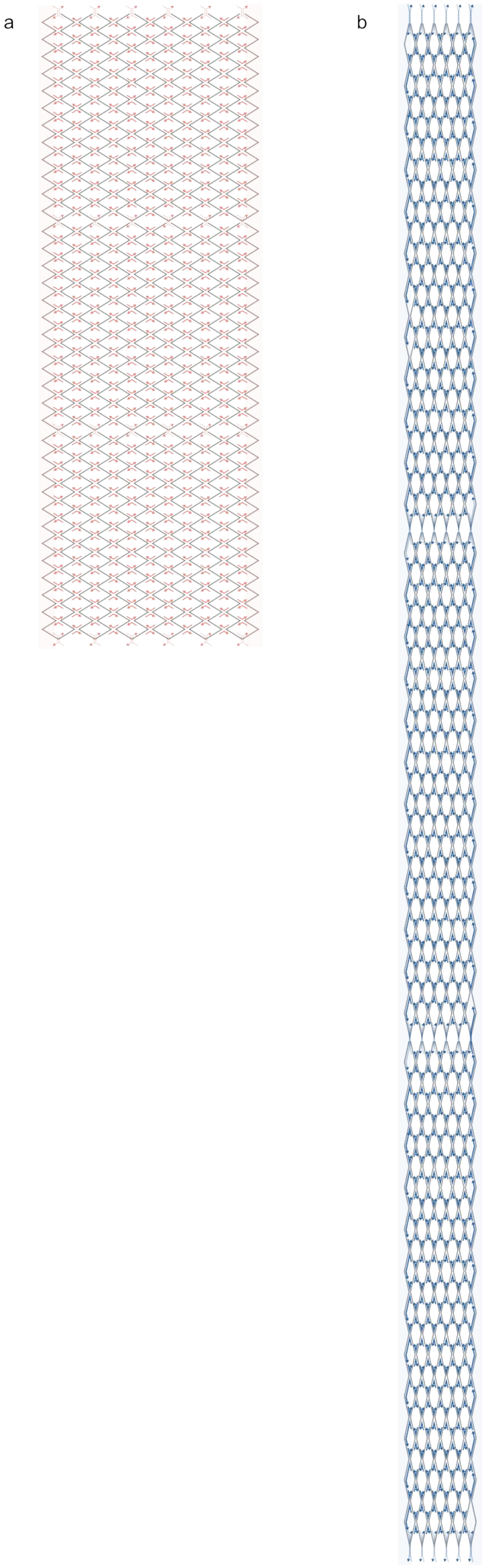

Figure S16. Schematic diagrams of conformations with strand level of details are provided in a (10B×24H rectangle multimer) and b (10H×24B rectangle multimer). The allosteric sites are highlighted. Staples to connect neighboring origami stuctures are shown in dashed lines.

1.3 Measurements of different allosteric states in DNA nanostructures

Tables S1 and S2 show the measurements of allosteric states for individual DNA nanostructures. The lengths of both long side and short side are measured for the rectangles. Diameters and/or heights are measured for tubes and disc. Widths are measured for ribbons. All measurements were performed using ImageJ software.

Table S1. Measurement statistics of rectangles.

Rectangle	Conformation	Short side/ nm	Long side/ nm	N
25H×10B/ 25B×10H	Fat	76±7	92±8	30
	Thin	37±4	222±11	30
8B×31H/ 8H× 31B	Fat	67±10	125±10	30
	Thin	30±6	260±23	30
22H×11B/ 22B×11H	Fat	74±8	79±9	30
	Thin	41±6	180±17	30
10B fat ribbon / 10H thin ribbon	Fat	97±10 nm	N/A	30
	Thin	31±5 nm	N/A	30

Table S2. Measurement statistics of tubes and disc.

Structure	Conformation	Height/ nm	Diameter/ nm	N
22H×11B tube/ 22B×11H tube	Fat	86±5	21±3	12
	Thin	N/A	61±4	18
20H ×13B tube/ 20B×13H disc	tube	100±10	44±7	30
	disc	N/A	78±10	30

2. Results of stacking strength with different types of effector staples

2.1 Stacking strength gradient at allosteric sites with different types of effector staples

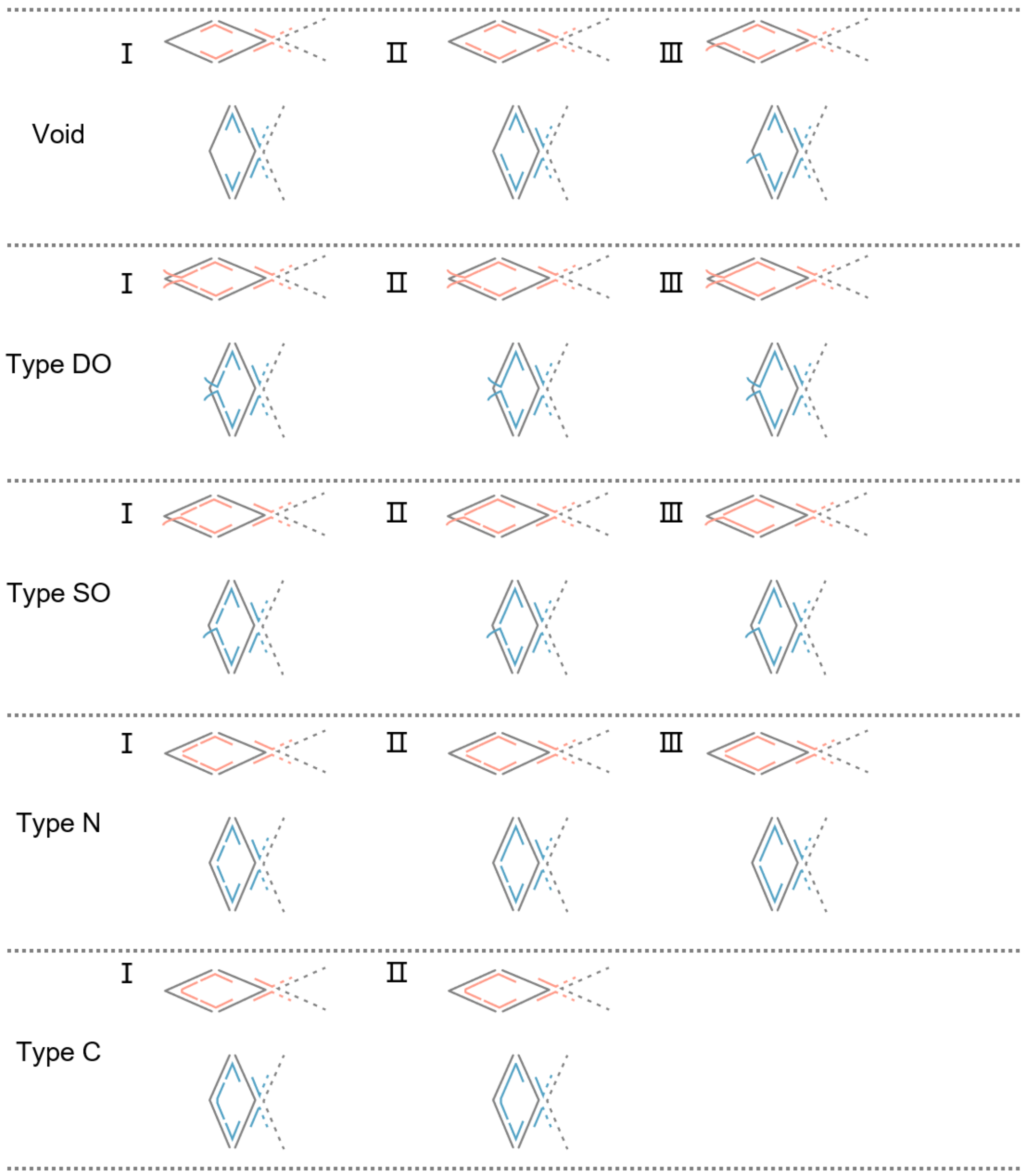

Figure S17. Classification of different main types of effector staples into subtypes. From top to bottom: void allosteric sites, type DO, type SO, type N and type C effector staples. Each subtype corresponds to a specific staple placement pattern. For example, void I can turn to type C II with DNA polymerase treatment.

2.2 Sub-classification of different types of effector staples

Figure S18 shows the full-size AFM images of the 25H×10B/25B×10H structures with different types of effector staples and Table S3 shows statistics of allosteric state distributions.

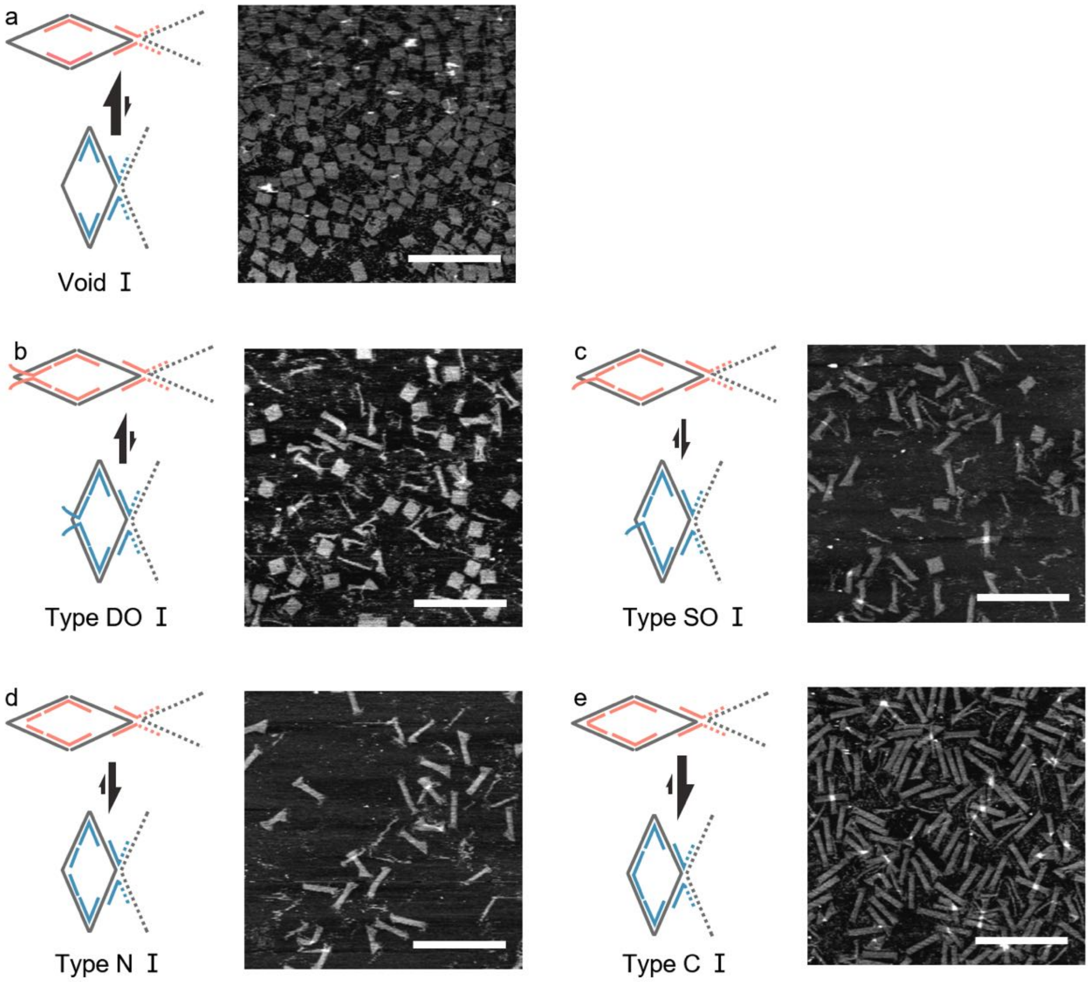

Figure S18. Stacking strength gradient of different types of effector staples. a. Void allosteric sites. b. Type DO effector staples. c. Type SO effector staples. d. Type N effector staples. e. Type C effector staples. Left: strand diagrams of effector staples at allosteric sites; right: AFM images. Scale bars: 600 nm.

Table S3. Gradient stacking strengths of different types of effector staples.

Effector staple type	Distribution			
	Fat%	Broom%	Thin%	N
Void	100	0	0	168
Type DO	74	24	2	482
Type SO	16	61	23	252
Type N	0	57	43	258
Type C	0	7	93	480

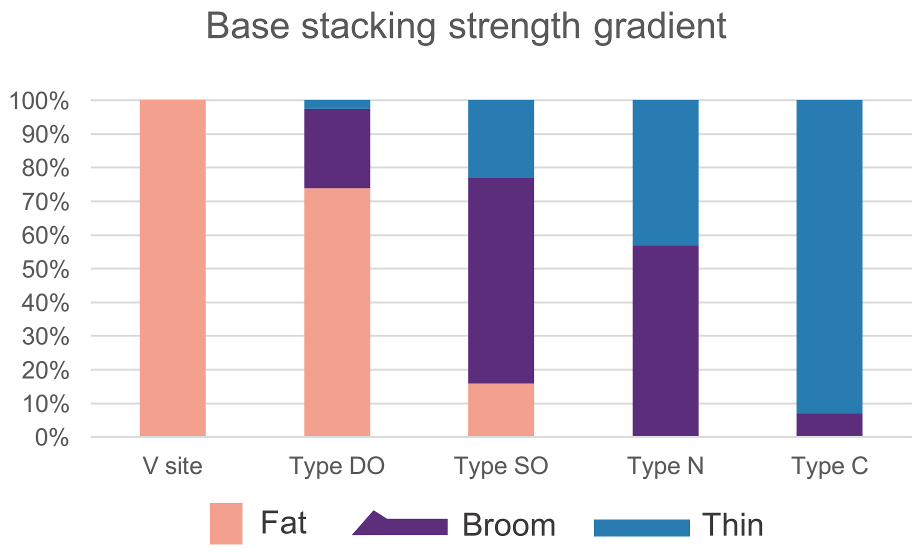

2.3 Calculation of free energies at allosteric sites with different types of effector staples

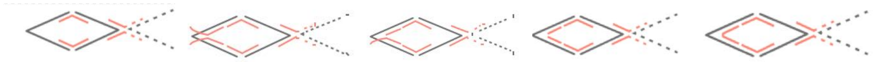

	Void	Type DO	Type SO	Type N	Type C
**Hybridization**	–	-672.4 Kcal/mol	-672.4 Kcal/mol	-672.4 Kcal/mol	-753.8 Kcal/mol
**Stacking**	–	-31.6 Kcal/mol	-31.6 Kcal/mol	-31.6 Kcal/mol	–
**Penalties**	–	74.4 Kcal/mol	33.6 Kcal/mol	–	–

ΔG	-629.6 Kcal/mol	-40.8 Kcal/mol	-33.6 Kcal/mol	-49.8 Kcal/mol

Figure S19. Calculation of free energies at allosteric sites with different types of effector staples. The free energy values of base-stacking(1), hybridization/initiation(2) and overhang thermodynamic penalty(3) are used for estimating the free energy difference of different conformations.

The structure with type DO effectors has a ΔG = -629.6 Kcal/mol over the structure with void allosteric sites. Although we assume that only a small portion of the energy difference contributes to the conformational change but it is still challenging how to extract the specific portion. The ΔG of structure with type SO effectors over structure with type DO effectors is -40.8 Kcal/mol; the ΔG of structure with type N effectors over that with type SO effectors is -33.6 Kcal/mol; the ΔG of structure with type C effectors over that with type N effectors is -49.8 Kcal/mol. The calculation of these three pairs better describes the energetic contribution of the conformational change since they all have the same number base pairs.

3. Results of allostery transition map with different enzyme treatments

As shown in Figures S20 to S27, exonuclease (Figure S20 and S21), ligase (Figures S22 to S24) and polymerase (Figures S25 and S27) were employed to drive the allosteric transition. Distributions of allosteric states without and with enzyme treatments are shown in Table S4.

3.1 Allosteric transition with exonuclease

We modified type SO effector staples to type N effector staples by exonuclease (Figure S20-21).

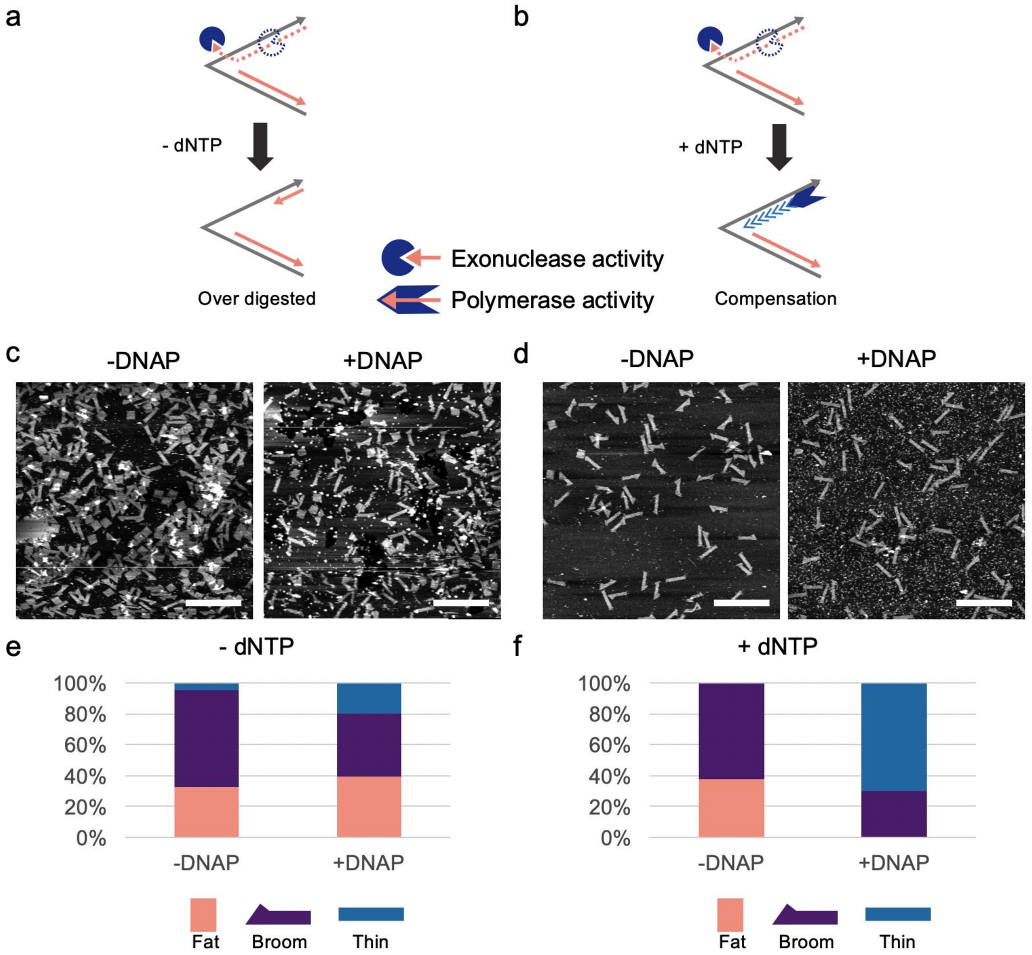

Figure S20. Digestion from type SO effector staples to type N effector staples by exonuclease. This control experiment verifies that polymerase activity is required for compensated digestion. a. Working model of over digestion. The 3’ to 5’ exonuclease activity of Q5 DNA polymerase leads to an over digestion of effector staples in the absence of dNTP supplement (without compensation by DNA polymerase elongation). b. Working model of compensated digestion. The 5’ to 3’ polymerase activity compensates the over digested effector staple in the presence of dNTP. c and e. AFM results (c) and distributions (e) of allosteric transition without (left) and with (right) Q5 DNA polymerase in the absense of dNTP supplment. d and f. AFM results (c) and distributions (e) of allosteric transition without (left) and with (right) Q5 DNA polymerase in the presence of dNTP supplment. Samples with and without Q5 DNA polymerase were incubated at 37 °C for 5 h before imaging. Scale bars: 600nm.

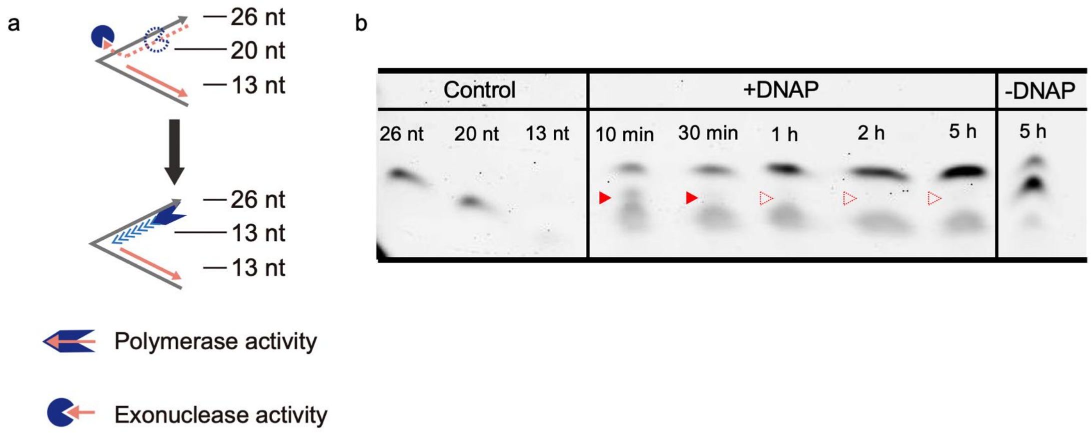

Figure S21. Verification of exonuclease activity of Q5 DNA polymerase. a. Working model of DNA exonuclease treatment. The lengths of each strand are specified. b. Polyacrylamide gel electrophoresis results of exonuclease treatment. Disappearance of the 20 nt band over the time course indicates a proper digestion of the single-stranded overhang (from 20 nt to 13 nt by the exonuclease activity of Q5 DNA polymerase). Samples with Q5 DNA polymerase were incubated at 37°C for 10 min, 30 min, 1 h, 2 h and 5 h before characterization, and sample without Q5 DNA polymerase was incubated at 37°C for 5 h before characterization. The solid triangles point at 20 nt bands and the hollow triangles point at the disappearing 20 nt bands.

3.2. Allosteric transition with ligase

We modified type N effector staples to type C by T4 ligase. Because of the substantial stacking strength of type N effector staples, the type N to type C conversion did not show a significant allosteric transition (Figure S22). Therefore, we only placed 1/3 of the N type effector staples to maintain a fat rectangle conformation initially. Then an allosteric transition to thin rectangle was resulted from T4 ligase treatment (Figure S23).

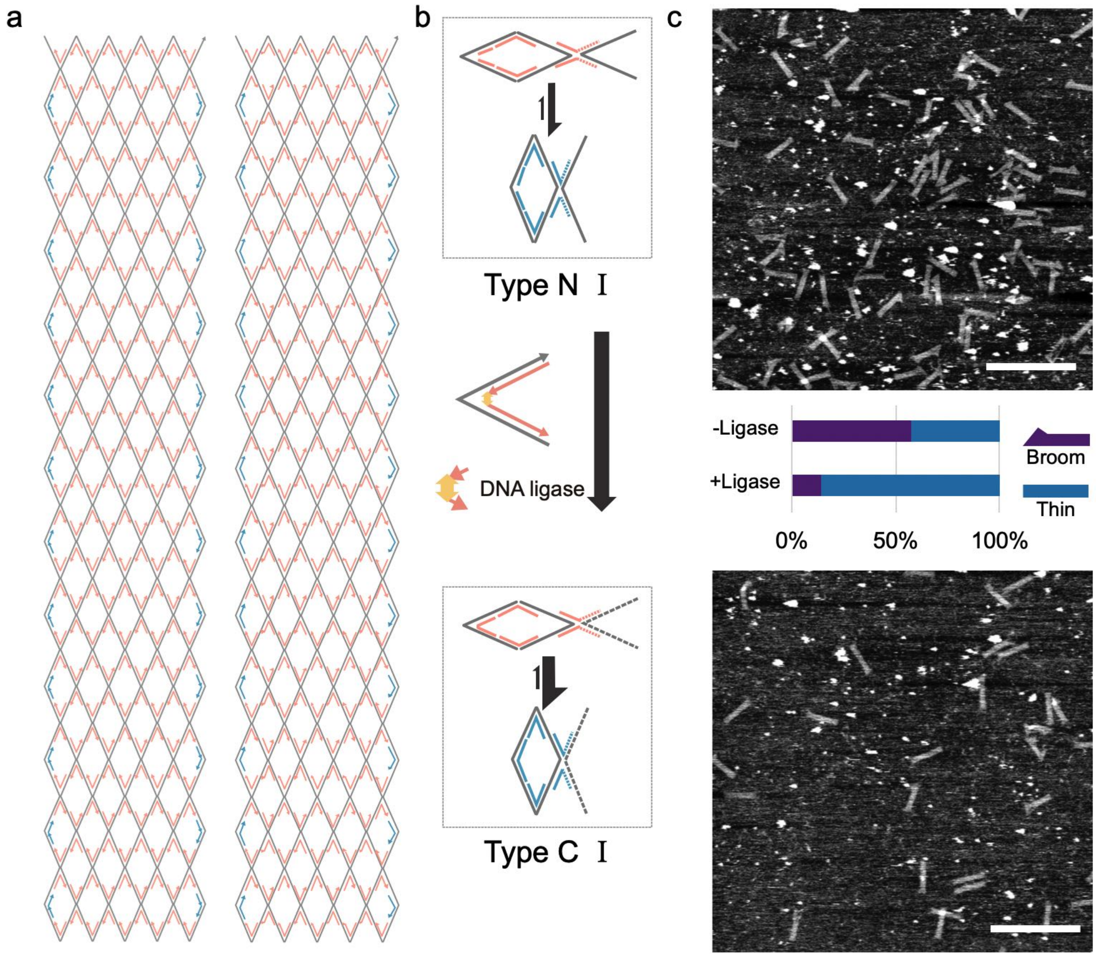

Figure S22. Nick sealing from type N effector staples to type C effector staples by ligase a. Schematic diagrams of 10B×25H and 10H×25B rectangles with all allosteric sites placed with effector staples. The type N staples (left) turn into type C staples (right) by ligation. b. Working model of nick sealing with DNA ligase treatment. c. Results of the allosteric transition from 10B×25H to 10H×25B based on DNA ligase treatment. Top: AFM result without enzyme treatment. Middle: distributions of allosteric states without and with enzyme treatment. Bottom: AFM result with enzyme treatment. Samples with and without T4 DNA polymerase treatment were incubated at 16 °C for 17 h before imaging. Scale bars: 600 nm.

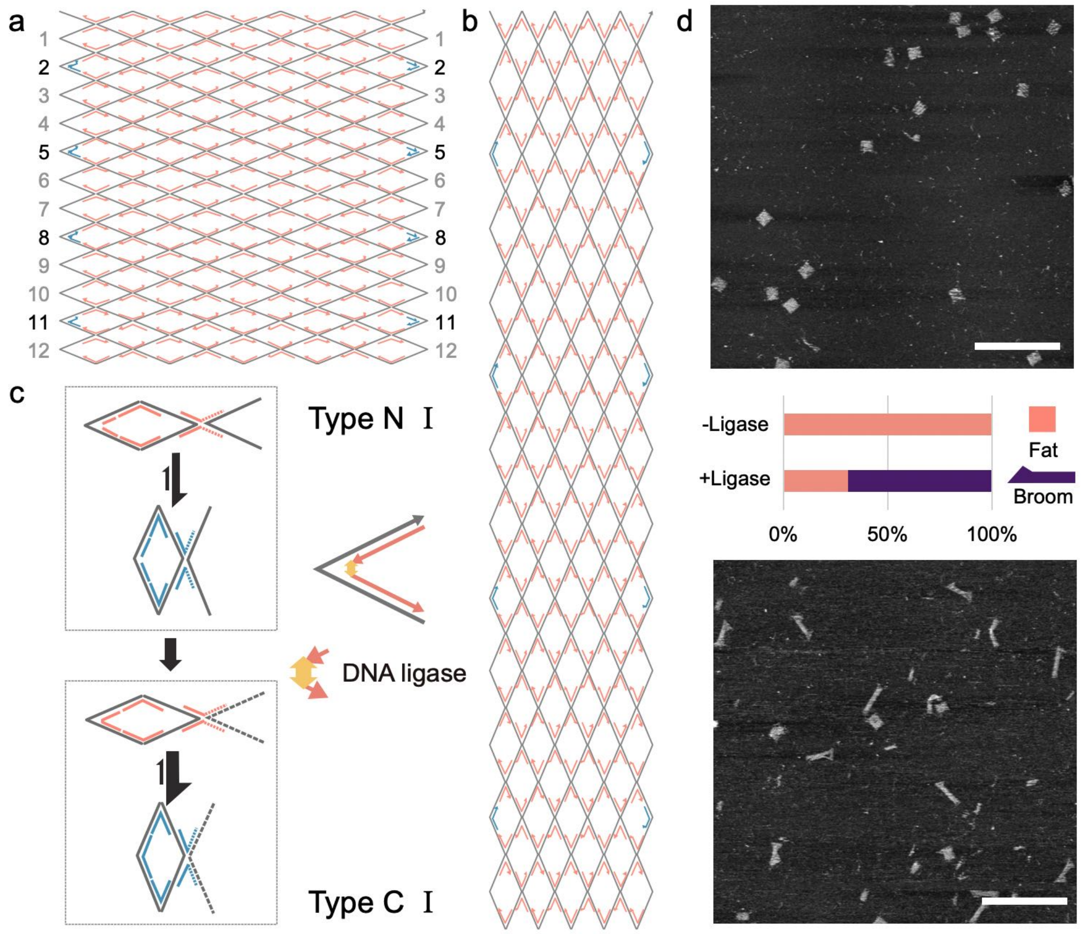

Figure S23. Nick sealing from type N effector staples to type C effector staples by ligase at chosen allosteric sites. a. Schematic diagrams of 10B×25H rectangle with only 8 sites (row numbers highlighted) of the total 24 allosteric sites placed by type N staples. b. Schematic diagrams of 10H×25B rectangle with chosen allosteric sites placed by type C staples. c. Working model of nick sealing with DNA ligase treatment. c. Results of the allosteric transition from a 10B×25H to a 10H×25B based on DNA ligase treatment. Top: AFM result without enzyme treatment. Middle: distributions of allosteric states without and with enzyme treatment (details in table S4). Bottom: AFM result with enzyme treatment. Scale bars: 600 nm.

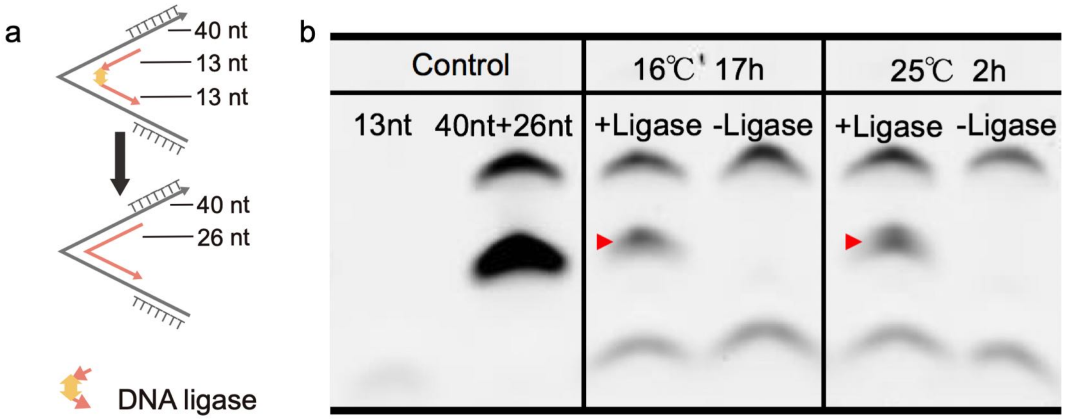

Figure S24. Verification of nick sealing of T4 ligase. a. Working model of T4 ligase treatment. The lengths of each strand are specified. b. Polyacrylamide gel electrophoresis results of exonuclease treatment. Appearance of the 26nt band indicates an efficient sealing of the nicks (from two 13 nt strands to one 26 nt strand due to the ligation). Samples with and without T4 ligase were incubated at 16 °C for 17h or 25°C for 2h before characterization.

3.3. Allosteric transition with polymerase

We modified void allosteric sites to type SO effector staples (Figure S25), type N effector staples (Figure S26), and type C effector staples (Figure S27) by DNA polymerase.

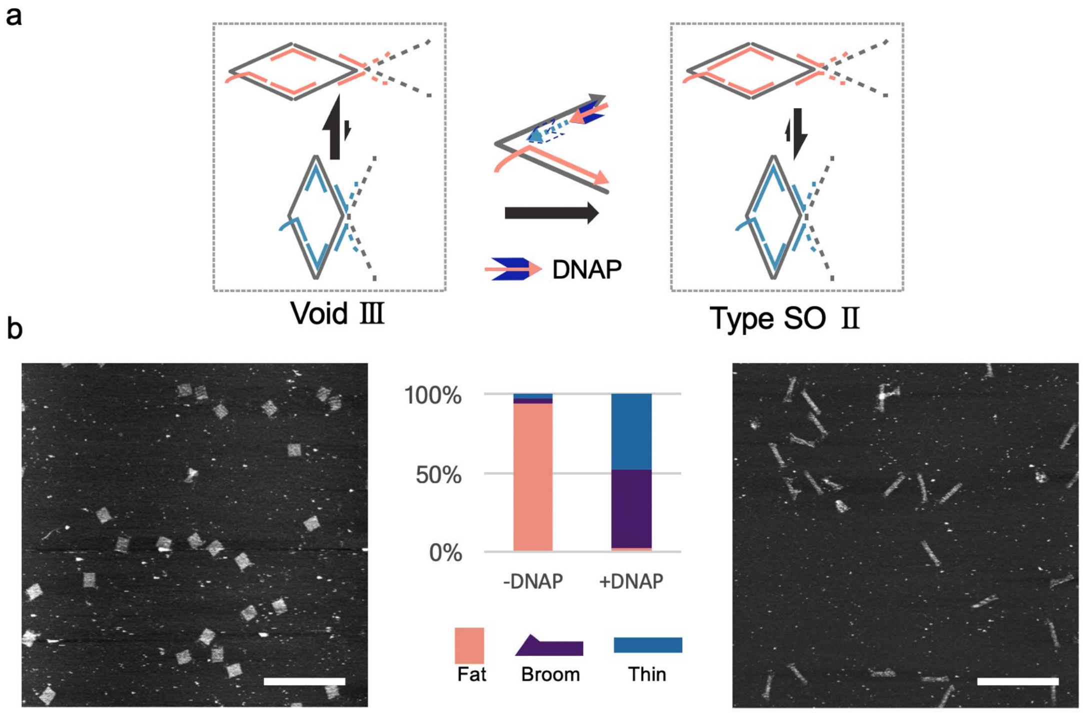

Figure S25. Gap filling from void allosteric sites to type SO effector staples by DNA polymerase. a. Working model of allosteric transition based on DNA polymerase treatment. b. AFM results of the rectangle distribution without (left) and with (right) Q5 DNA exonuclease treatment. The corresponding distributions of allosteric states are shown in the middle (details in table S4). Samples with and without Q5 DNA polymerase were incubated at 37 °C for 5 h before imaging. Scale bars: 600 nm.

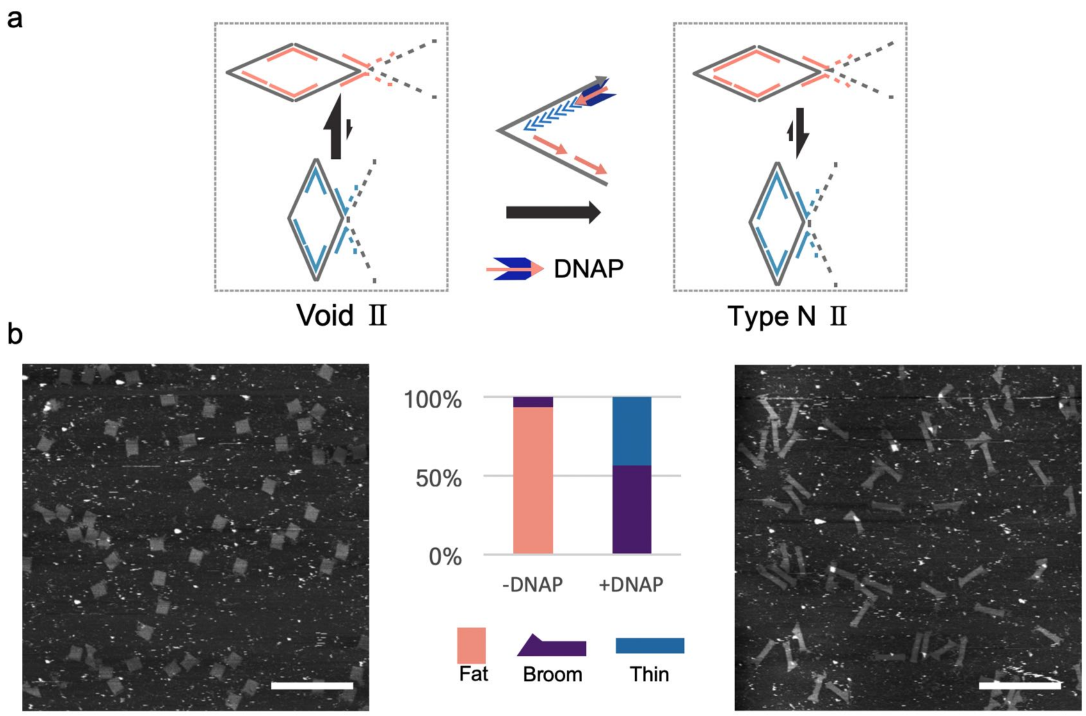

Figure S26. Gap filling from void allosteric sites to type N effector staples by DNA polymerase. a. Working model of allosteric transition based on DNA polymerase treatment. b. AFM results of the rectangle distribution without (left) and with (right) Q5 DNA exonuclease treatment. The corresponding distributions of allosteric states are shown in the middle (details in table S4). Samples with and without Q5 DNA polymerase were incubated at 37 °C for 5 h before imaging. Scale bars: 600 nm.

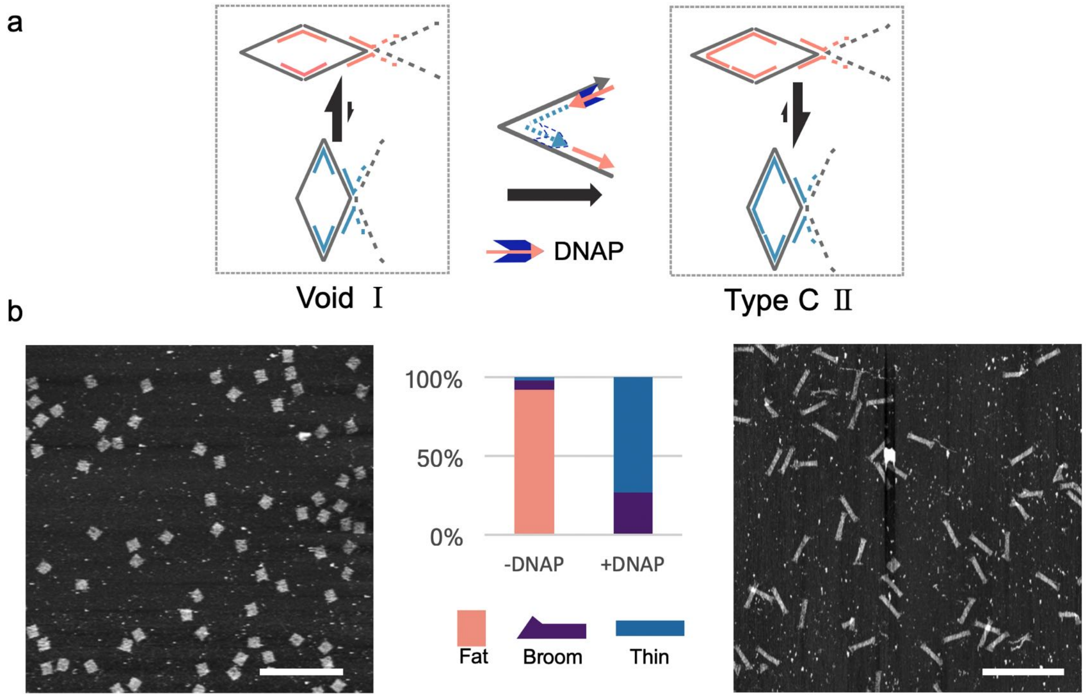

Figure S27. Gap filling from void allosteric sites to type C effector staples by DNA polymerase. a. Working model of allosteric transition based on DNA polymerase treatment. b. AFM results of the rectangle distribution without (left) and with (right) Q5 DNA exonuclease treatment. The corresponding distributions of allosteric states are shown in the middle (details in table S4). Samples with and without Q5 DNA polymerase were incubated at 37 °C for 5 h before imaging. Scale bars: 600 nm.

3.4 Allosteric states distribution without and with enzyme treatment

Table S4. Distributions of allosteric states without and with enzyme treatment.

Modification	Enzyme	Distribution without treatment				Distribution with treatment			
		Fat%	Broom%	Thin%	N	Fat%	Broom%	Thin%	N
V Ⅲ to SO Ⅱ	Q5 DNAP	94	3	3	193	2	50	48	124
SO I to N I	Q5 DNAP (exo)	37	63	0	221	0	30	70	177
V II to N II	Q5 DNAP	94	6	0	321	1	55	43	124
N I to C I	T4 ligase	100	0	0	60	31	69	0	439
V I to C II	Q5 DNAP	92	6	2	182	1	26	73	279
V I to C II	T4 DNAP	95	0	5	532	71	1	28	175
SO I to N I	Q5 DNAP (exo, no dNTP)	32	63	5	197	39	41	20	102

References

1. Protozanova, E., Yakovchuk, P. and Frank-Kamenetskii, M.D. (2004) Stacked–unstacked equilibrium at the nick site of DNA. *Journal of molecular biology*, **342**, 775-785.
2. SantaLucia, J. (1998) A unified view of polymer, dumbbell, and oligonucleotide DNA nearest-neighbor thermodynamics. *Proceedings of the National Academy of Sciences*, **95**, 1460-1465.
3. Srinivas, N., Ouldridge, T.E., Šulc, P., Schaeffer, J.M., Yurke, B., Louis, A.A., Doye, J.P. and Winfree, E. (2013) On the biophysics and kinetics of toehold-mediated DNA strand displacement. *Nucleic acids research*, **41**, 10641-10658.
